# Topological near fields generated by topological structures

**DOI:** 10.1126/sciadv.abq0910

**Published:** 2022-10-14

**Authors:** Jie Peng, Ruo-Yang Zhang, Shiqi Jia, Wei Liu, Shubo Wang

**Affiliations:** ^1^ Department of Physics, City University of Hong Kong, Tat Chee Avenue, Kowloon, Hong Kong, China.; ^2^ Department of Physics, The Hong Kong University of Science and Technology, Clear Water Bay, Kowloon, Hong Kong, China.; ^3^ College for Advanced Interdisciplinary Studies, National University of Defense Technology, Changsha, Hunan 410073, China.; ^4^ City University of Hong Kong Shenzhen Research Institute, Shenzhen, Guangdong 518057, China.

## Abstract

The central idea of metamaterials and metaoptics is that, besides their base materials, the geometry of structures offers a broad extra dimension to explore for exotic functionalities. Here, we discover that the topology of structures fundamentally dictates the topological properties of optical fields and offers a new dimension to exploit for optical functionalities that are irrelevant to specific material constituents or structural geometries. We find that the nontrivial topology of metal structures ensures the birth of polarization singularities (PSs) in the near field with rich morphologies and intriguing spatial evolutions including merging, bifurcation, and topological transition. By mapping the PSs to non-Hermitian exceptional points and using homotopy theory, we extract the core invariant that governs the topological classification of the PSs and the conservation law that regulates their spatial evolutions. The results bridge singular optics, topological photonics, and non-Hermitian physics, with potential applications in chiral sensing, chiral quantum optics, and beyond photonics in other wave systems.

## Teaser

The topological properties of optical near fields are solely dictated by the overall topology of optical structures.

## INTRODUCTION

The concept of topology has provided new perspectives for physicists to explore unconventional properties of physical systems, such as the one-way edge states in topological insulators and their counterparts in classical wave systems that have attracted considerable interest recently (*1*). These properties are associated with the topology of the momentum space (i.e., Brillouin zone). Topology in the real space can also give rise to intriguing physical properties and phenomena. In particular, Maxwell’s equations allow unique solutions with nontrivial topology in the real space, where the field lines, phase singularities (i.e., optical vortices), or polarization disclinations can form links, knots, and toroids (*2*–*12*). These topological configurations of electromagnetic fields can enable highly flexible manipulations of phase and polarization with unprecedented precision for various applications.

At an arbitrary point of the three-dimensional (3D) real space, the end of electric/magnetic field vector of a generic monochromatic electromagnetic wave traces out an ellipse, i.e., the field is elliptically polarized. The distribution of the polarization ellipses can form topological defects known as polarization singularities (PSs) (*13*), which include C points (where the field is circularly polarized and the direction of the major axis of polarization ellipse is ill-defined), L points (where the field is linearly polarized and the normal direction of polarization ellipse is ill-defined), and V points (where the field norm is zero and the field direction is ill-defined). The 3D lines formed of the PSs are referred to as C lines, L lines, and V lines accordingly. These PS lines (PSLs) can emerge during light focusing (*14*), scattering (*15*–*19*), and interference (*7*, *20*), in nanostructures including metasurfaces (*21*, *22*) and photonic crystals (*23*). The integration of singularity and topology theory has revealed richer physics including geometric phases (*24*, *25*), bound states in the continuum (BIC) (*26*–*28*), Hermitian topological nodal degeneracies (*29*, *30*), non-Hermitian exceptional points (EPs) (*31*–*35*), etc.

The explicit geometry of optical structures decides the local resonance of optical modes and gives rise to novel optical devices and wave functional materials, such as nanoantennas (*36*), metamaterials (*37*), and metasurfaces (*38*). As a result, conventional studies in photonics mainly focus on the geometry and rarely pay attention to the overall topology of the structures investigated. It thus becomes interesting to ask: What optical properties are determined solely by the overall topology of optical structures? How can the topology of optical polarization fields and the topology of optical structures be interconnected?

Here, we establish a universal and exact connection between the topology of optical fields and that of optical structures, revealing how the births and topological evolutions of magnetic PSs in the near fields are bounded by the topology and symmetry of the structures. As is required by the Poincaré-Hopf theorem, the existence of the PSs is topologically protected, and they are characterized by quantized topological indices with the index sum solely decided by the genus of the structures. We further demonstrate that, by incorporating extra spatial symmetries (such as the mirror symmetry and the generalized rotational symmetry) for the structures and the incident fields, higher-order PSs/PSLs and topologically stable nexuses of PSLs can emerge, around which the polarizations evolve into notable configurations such as mirror-symmetric double-twist Möbius strips (*39*, *40*). To grasp the underlying invariant properties of the PSs in the continuous evolutions (e.g., merging, bifurcation, and topological transition), we map the real-space C points to the EPs of a two-band non-Hermitian Hamiltonian, upon which homotopy theory can be directly used to identify the core invariant to classify all topological evolutions.

## RESULTS

### PSs protected by surface topology of structures

A general monochromatic magnetic field can be expressed as **H** = (**A** + *i***B**)*e*^*i*θ^, where **A** and **B** are the major and minor axes of the polarization ellipse, and θ = Arg(Ψ)/2 with Ψ = **H** · **H**. The C points of the magnetic field **H** correspond to the phase singularities of the scalar field Ψ and generally can form stable C lines in 3D space without any symmetry protection. The C line can be characterized by two topological indices (i.e., winding numbers): the polarization index *I*_pl_ = 1/(4π) ∮ *d*φ and the phase index *I*_ph_ = 1/(2π) ∮ ∇Arg(Ψ) · *d***r**, where φ is the azimuthal angle on the Poincaré sphere for the in-plane polarization (i.e., in the plane of the polarization ellipse at a C point) and both integrals are evaluated on a small loop enclosing the C line (*25*, *41*). We note that *I*_pl_ is uniquely defined at each C point provided that the magnetic spin **S** = Im(H^*^ × **H**) is not normal to the C line. At the points where **S** is normal to the C line, *I*_pl_ may change sign, making *I*_pl_ not a global invariant along the C line. In contrast, although the sign of *I*_ph_ depends on the direction of the integration loop, it is invariant against continuously moving the loop along the C line. Therefore, *I*_ph_ can endow the C line with a positive direction **t**_c_ = sign(*I*_ph_)**t**, where **t** is the tangent vector of the C line complying with the right-hand rule of the integration loop of *I*_ph_ (*41*). One can prove that the two indices of a C point are related by *I*_pl_ = sign(**t** · **S**)*I*_ph_/2 (see the Supplementary Materials).

**Fig. 1.**
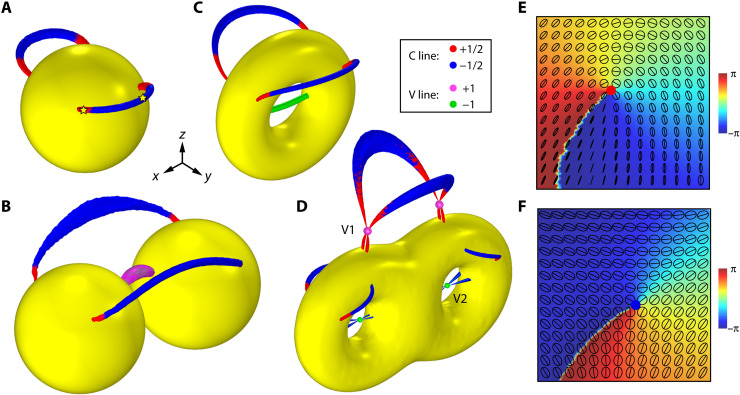
Polarization singularities generated by topological structures. (**A** to **D**) The C lines (blue/red) and V lines (magenta/green) generated by metal structures (yellow) with Euler characteristic χ = +2, +4,0, −2. The small magenta and green spheres in (D) denote the V points formed by crossings of C lines. (**E** and **F**) Polarization ellipses and Arg(Ψ) on a plane for the C points marked by the stars in (A). The planes are perpendicular to the local spin at the C points. The line segments in the ellipses denote the polarization major axes. The colors show the value of Arg(Ψ).

Let us first consider a metal sphere under the incidence of a plane wave propagating in *z* direction and the magnetic field is linearly polarized in *y* direction. Without loss of generality, we assume that the metal is gold characterized by a Drude model (see Materials and Methods). We conducted full-wave numerical simulations of the system by using a finite-element package COMSOL Multiphysics. Here and in what follows, we consider the PSLs emerging in the total magnetic field. The numerically obtained PSLs at the frequency *f* = 100 THz are shown in Fig. 1A. A pair of C lines emerge near the surface of the sphere. For each C line, the polarization index *I*_pl_ changes from +1/2 (red) to −1/2 (blue) and then back to +1/2. Such sign flips happen at the points where **S** ⊥ **t**_c_. In Fig. 1 (E and F), we show the polarization ellipses on a plane perpendicular to **S** for the C points marked by the yellow stars in Fig. 1A, where the line segments inside the polarization ellipses denote the major axes **A** and the red (blue) dot denotes the C point with index *I*_pl_ = +1/2 (*I*_pl_ = −1/2). The phase Arg(Ψ) is also shown on the same planes, which clearly carries the phase index *I*_ph_ = +1 and *I*_ph_ = −1, correspondingly, following the relationship *I*_pl_ = *I*_ph_/2. The emergence of such a configuration of C lines is puzzling, and it becomes even intriguing for two coupled metal spheres, as shown in Fig. 1B. Under the incidence of the same plane wave, two C lines with *I*_pl_ = +1/2 appear near the surfaces and connect the two spheres. In addition, a V line (magenta-colored) appears in the gap region between the spheres due to the accidental degeneracy of a pair of C lines with the same polarization index *I*_pl_ = +1/2. We thus can define its index as *I*_pl_ = (+1/2) × 2 = +1 by summing up *I*_pl_ of the two superposed C lines. Although a V line cannot stably exist in general, the intersections of two C lines can form stable V points protected by mirror symmetry, as will be proved later.

We now ask the question: What determines the morphologies of the PSLs and their topological indices? It turns out that the answer is rooted in the topological properties associated with the geometry of the metal spheres. Under the excitation of the incident electromagnetic field, currents are induced in metal structures. These currents mainly localize within a thin surface layer of the structures, the thickness of which is approximately equal to the “skin depth” (i.e., the depth that light penetrates the metal). By the boundary conditions, the magnetic field **H** near the metal surface is dominated by its tangent component (see the Supplementary Materials). Consequently, the major axis **A** of the polarization ellipses can be considered a tangent bivector field (i.e., line field) defined on a 2D smooth manifold *M* (i.e., the surfaces of the structures). According to the Poincaré-Hopf theorem for tangent line fields, a finite number of isolated singularities of this field must emerge on the surfaces of the structures (*42*). These singularities are exactly the C points and/or V points, and the sum of their topological polarization indices must satisfy ∑**r**_s_*I*_pl_(**r**_s_) = χ(*M*), where **r**_s_ denote the PSs on the smooth manifold *M*, and χ denotes the Euler characteristic of *M*. For a closed orientable surface, χ is directly given by the genus *g* of the surface: χ = 2 − 2*g* (*42*). For the single sphere case in Fig. 1A, there are four C points on the sphere’s surface with the same index *I*_pl_ = +1/2, thus, ∑*I*_pl_ = (+1/2) × 4 = +2 = χ, consistent with the Poincaré-Hopf theorem. For the coupled spheres in Fig. 1B, there are four C points and two V points on the spheres’ surfaces with total index ∑*I*_pl_ = (+1/2) × 4 + 1 × 2 = +4 = χ, again satisfying the theorem.

To further verify the above interpretations based on Poincaré-Hopf theorem, we calculated the PSLs generated by metal structures with genus *g* = 1 and *g* = 2 as shown in Fig. 1 (C and D), respectively. The same incident plane wave is applied in Fig. 1 (A to D), and we focus on the frequency range with small skin depth. Under this condition, the physics is general and is not restricted to a particular frequency or metal material. For the single torus in Fig. 1C, in addition to two C lines, we observe an accidental V line with *I*_pl_ = −1 (green-colored) emerging inside the hole, corresponding to a pair of degenerate C lines with *I*_pl_ = −1/2. The index sum of the PSs for the single torus is ∑*I*_pl_ = (+1/2) × 4 + (−1) × 2 = 0, which is equal to the Euler characteristic of a torus. Figure 1D shows the PSLs generated by the double torus with genus *g* = 2. A rich structure of the PSLs appears with crossings of C lines. There are totally twenty C points on the metal surface with total index ∑*I*_pl_ = (+1/2) × 8 + (−1/2) × 12 = −2, again agreeing with the Euler characteristic of the double torus. These results establish a direct relation between the topology of optical structures and the topological properties of optical near fields. This relation holds for arbitrary metal structures as long as their geometric surfaces are smooth and the skin depth is small.

**Fig. 2.**
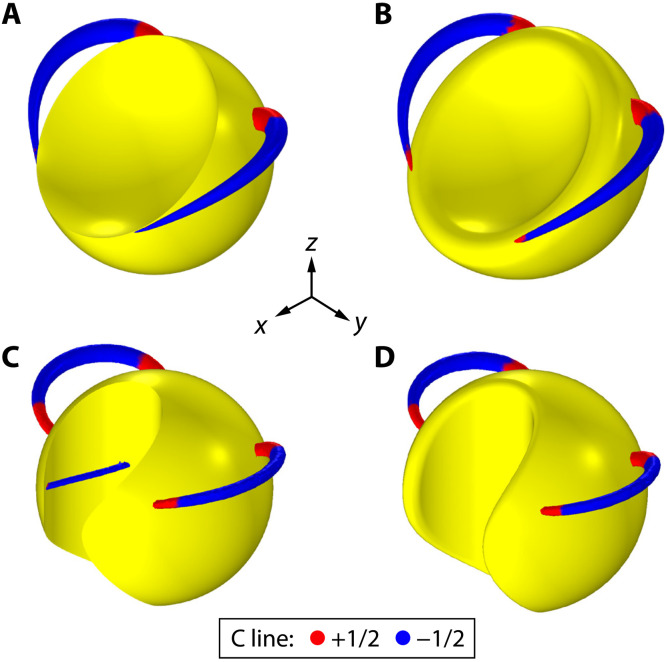
Effect of singular edges. (**A** and **B**) The C lines generated by a sphere with sharp circular edge and with the sharp edge smoothed out, respectively. (**C** and **D**) The C lines generated by a sphere with sharp irregular edge (created by removing a cylindrical portion from the sphere) and with the sharp edge smoothed out, respectively. There are two C lines in (A), (B), and (D) and four C lines in (C).

Since the emergence of PSLs is protected by the topology of the structures, their global topological properties are robust against continuously varying the geometry of structures unless singular perturbations are introduced to the geometry. For example, if we introduce sharp edges into the sphere such that the radii of curvature near the edges are comparable with the skin depth, then the total index of the surface PSs can change, as shown in Fig. 2A, where ∑*I*_pl_ = 0. The presence of sharp edges (where the tangent planes and thus the tangent fields are ill-defined) renders the original Poincaré-Hopf theorem for smooth manifolds inapplicable to the surface. Thus, the total index is not necessarily determined by the genus of the geometry. On the other hand, if the sharp edges are “smoothed out,” as shown in Fig. 2B, the global topological properties of the PSs recover, i.e., their indices satisfy the theorem again. A second example is given in Fig. 2C, where a cylindrical portion is removed from the sphere. In this case, the sharp edge induces two additional C lines with *I*_pl_ = −1/2, and the total index of the C points on the surface is zero. After smoothing the edges, the total index recovers the value of +2, as shown in Fig. 2D.

### Mapping to non-Hermitian EPs

The PSLs evolve as they extend away from the surfaces of the structures, leading to merging, bifurcation, and topological transition in the 3D space. To understand these phenomena, we use a mapping from the C points (real-space singularities) to the non-Hermitian EPs (parameter-space singularities). We introduce an auxiliary two-band non-Hermitian Hamiltonian associated with the magnetic field, $H(r)=H(r)\cdot \vec{\sigma }$, where $\vec{\sigma }=({\hat{\sigma }}_{x},{\hat{\sigma }}_{y},{\hat{\sigma }}_{z})$ is the vector of Pauli matrices. The Hamiltonian has two eigenvalues $h_{\pm }=\pm \sqrt{H\cdot H}$ that are proportional to the eccentricity of the polarization ellipse (1 − ∣**B**∣^2^/∣**A**∣^2^) and are degenerate when the discriminant of the Hamiltonian’s characteristic polynomial reduces to zero: *D* = (*h*_+_ − *h*_−_)^2^ = 4**H** · **H** = 4Ψ = 0, i.e., the condition for the emergence of a C point or V point. Thus, the degeneracies of the non-Hermitian Hamiltonian, i.e., EPs and nondefective nodal points, just correspond to the C and V points of the magnetic field, respectively. This remarkable property allows a mapping from the topology of EPs given by the two-band non-Hermitian Hamiltonian to the topology of C points in magnetic field. By borrowing the topological classification of the two-band non-Hermitian Hamiltonian with separable bands (*43*), we can obtain the topological classification of the PSLs in the 3D real space. Specifically, in the absence of any symmetry constraint, the configuration space of the non-Hermitian Hamiltonian without degeneracies can be expressed by the coset space *X* ≃ (*S*^2^ × *S*^1^)/ℤ_2_ (see Materials and Methods) (*43*), which can be physically understood as the configuration space of the magnetic field in the real space: *S*^2^ stands for the orientation sphere of the major axis **A** of the polarization ellipse; *S*^1^ corresponds to the circle of the phase Arg(Ψ) = 2θ; ℤ_2_ denotes the redundancy that both the direction of **A** and *e*^*i*θ^ change sign simultaneously. We note that since only C and V points are of our interest, the vanishing of the minor axis **B** of the polarization ellipse (i.e., the condition of L points) is irrelevant to the topological classification here. The first homotopy group of the configuration space *X* gives the topological classification of the polarization field along any closed loop in the space (*43*): π_1_(*X*) = π_1_((*S*^2^ × *S*^1^)/ℤ_2_) = π_1_(*S*^1^/ℤ_2_) = ℤ, which essentially classifies the topologically different morphisms of the PSLs (i.e., C lines in general) encircled by the loop. The ℤ topological invariant distinguishing different homotopy equivalence classes in π_1_(*X*) is just the phase winding number of Ψ along a loop γ, i.e., phase index *I*_ph_(γ). It corresponds to the energy vorticity, also known as the discriminant number, in the notation of non-Hermitian physics (*33*–*35*). Therefore, the phase index *I*_ph_(γ) is conserved against continuous deformation of the loop; it is a complete index that can characterize all topological phases associated with C lines. In comparison, the polarization index *I*_pl_ defined along an arbitrary loop, characterized by the trivial or Möbius twists of the major axis **A**, is not a complete index (see the Supplementary Materials). As we have shown, if a loop only encloses one C line, then the phase index *I*_ph_ defined with the loop can assign a positive direction **t**_c_ to the C line. More broadly, the topological index *I*_ph_(γ) defined with an arbitrary loop γ counts the net number of directed C lines passing through the loop.

**Fig. 3.**
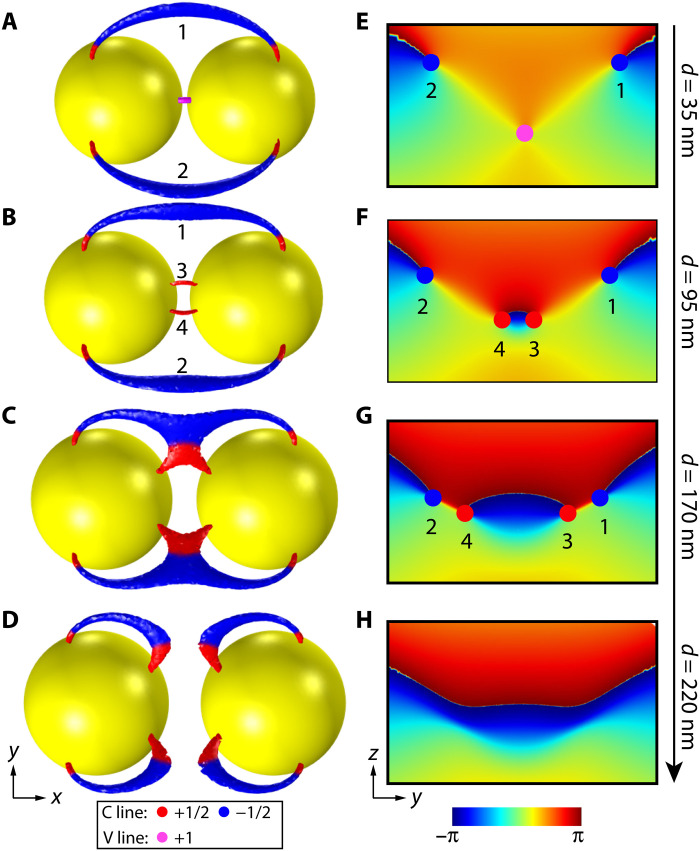
Topological transition of PSLs. (**A** to **D**) Topological transition of PSLs when two spheres are gradually separated. The accidental V line in the gap bifurcates into two C lines (labeled as 3 and 4) that further merge with rest C lines (labeled as 1 and 2) to enable the topological transition. (**E** to **H**) Arg(Ψ) on the *yz* mirror plane at different separations of the spheres corresponding to (A) to (D).

Since the topological invariant ℤ is equal to the phase index *I*_ph_, we can apply this invariant index to understand the topological transition of PSLs. Consider the double-sphere case in Fig. 1B, at large separation of the spheres, the PSLs must reduce to that of two isolated spheres (corresponding to two copies of Fig. 1A). This involves a topological transition. Figure 3 (A to D) shows the PSLs for the double-sphere case when the separation is increased, where we label the C lines by 1, 2, 3, and 4. Figure 3 (E to H) shows the phase Arg(Ψ) on the *yoz* plane in the middle of the two spheres for the cases of Fig. 3 (A to D). To approach the configuration in Fig. 3D, the V line with *I*_ph_ = 0 in Fig. 3A must bifurcate into two C lines with *I*_ph_ = ±1 that can merge with the rest C lines. This is indeed the configuration in Fig. 3 (B and F), where the V line bifurcates into two C lines 3 and 4 with phase indices *I*_ph_ = +1 and *I*_ph_ = −1, respectively. Merging and annihilation can only happen to a pair of C lines with opposite phase indices. This explains why C line 1 merges with C line 3, and C line 2 merges with C line 4, enabling the topological transition and opening a gap, as shown in Fig. 3 (C, D, G, and H). In addition, the results in Fig. 3 indicate that C lines with opposite polarization index *I*_pl_ do not necessarily annihilate because their chirality (i.e., spin **S**) can be different. An example is the C lines 1 and 4 in Fig. 3 (B and F), which have opposite *I*_pl_ and opposite chirality. On the other hand, this can be easily understood based on the phase index since both C lines carry *I*_ph_ = −1.

### Integration of topology and mirror symmetry

**Fig. 4.**
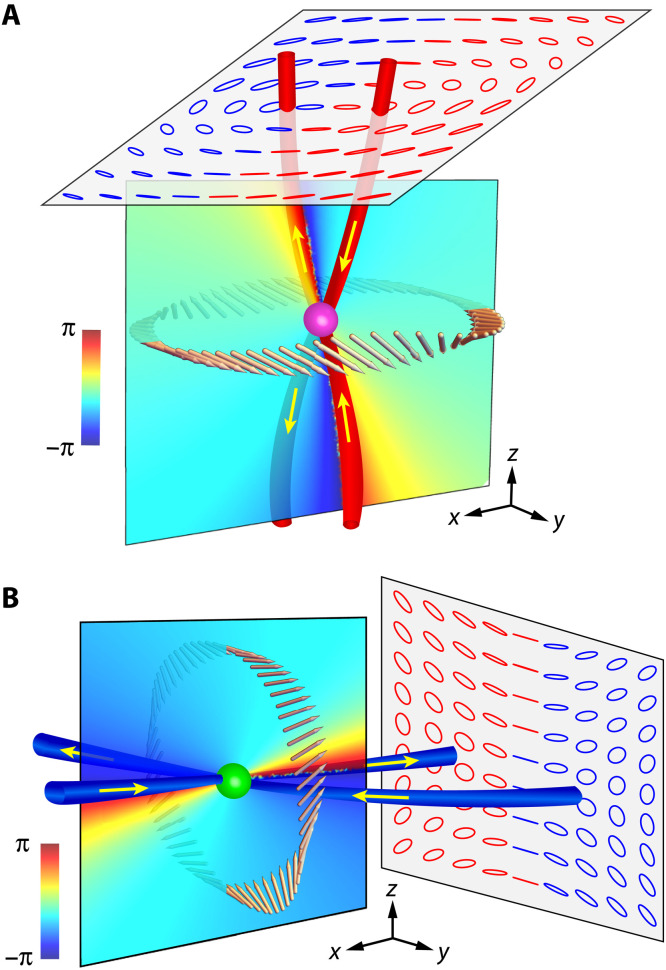
V points due to mirror symmetry. (**A**) V1 point due to the crossing of two C lines with *I*_pl_ = +1/2. (**B**) V2 point due to crossing of two C lines with *I*_pl_ = −1/2. The blue (red) color of the polarization ellipses corresponds to negative (positive) spin. The colors on the *xz* mirror plane at the V points show Arg(Ψ). The pink arrows in (A) and (B) denote the polarization major axes on a self-mirror symmetric loop, showing a double-twist Möbius strip. The yellow arrows show the directions of the C lines.

To further understand the properties of the PSLs in Fig. 1, it is necessary to discuss the combined effect of mirror symmetry and topology. If we impose a *y*-mirror symmetry about the mirror plane *y* = 0, marked as Π, then it is straightforward to show that the two-band Hamiltonian satisfies $H(r)=(-{\hat{m}}_{y}H({\hat{m}}_{y}r))\cdot \vec{\sigma }={\hat{\sigma }}_{y}H({\hat{m}}_{y}r){\hat{\sigma }}_{y},$ where ${\hat{m}}_{y}=\text{diag}(1,-1,1)$ denotes the mirror reflection operator for polar vectors about *y* = 0. Thus, the magnetic field in a mirror-symmetric system can be mapped to a Hamiltonian with a mirror symmetry. In this case, the magnetic field on the mirror plane Π only has perpendicular component, $H(x,0,z)=H_{y}(x,0,z)\hat{y}$; hence, the configuration space *X*_Π_ of the nonsingular magnetic field on Π is given by $X_{\Pi }=\{H=H_{y}\hat{y}|H_{y}\neq 0\}≃C-0≃S^{1}$. Therefore, we obtain the topological classification of the magnetic fields along the loops in Π according to the first homotopy group: π_1_(*X*_Π_) = π_1_(*S*^1^) = ℤ, which is characterized by the different phase winding number of *H_y_* along the loops. In this case, topologically stable V points just appear at the phase singularities of *H_y_* in the mirror plane. The phase index of each V point defined on a loop encircling the V point in the mirror plane must be double quantized: $I_{\text{ph}}=(1/2\pi )\oint \nabla \text{Arg}(H_{y}^{2})\cdot dr=(1/\pi )\oint \nabla \text{Arg}(H_{y})\cdot dr=\pm 2$. The conservation of the phase index *I*_ph_ = ±2 indicates that such a mirror symmetry–protected V point is not an isolated singularity but manifests as the intersection point of two C lines that pierce the mirror plane from the same side (see Fig. 4).

We now apply the above results to understand the V points labeled as V1 and V2 in Fig. 1D, which are nexuses of two mirror-partner C lines protected by a *y*-mirror symmetry of the system. Figure 4A shows the polarization ellipses and the phase Arg(Ψ) near V1 on a cutting plane and the *y* mirror plane, respectively. The polarization ellipses indicate that the two C lines have opposite chirality $S({\hat{m}}_{y}r)=-{\hat{m}}_{y}S(r)$ (denoted by the blue and red colors of the ellipses) but same polarization index *I*_pl_ = +1/2 (denoted by the red color of the C lines), as guaranteed by the mirror symmetry (see the Supplementary Materials). As a result, the two C lines are oppositely oriented about the mirror plane, i.e., $t_{c}(r_{c})=-t_{c}({\hat{m}}_{y}r_{c}$) according to the relation **t**_c_ = sign(*I*_pl_**S** · **t**)**t**, as shown by the yellow arrows on the C lines. In other words, both **S** and **t**_c_ behae as pseudo-vectors under mirror reflection. The phase Arg(Ψ) on the *y* mirror plane has a −4π variation around the V1 point, corresponding to a phase index *I*_ph_ = −2 (viewed from $+\hat{y}$ direction) and in accordance with the fact that the two C lines both point inward ($-\hat{y}$ direction) from the mirror plane at the V1 point. Figure 4B shows the polarization ellipses and the phase Arg(Ψ) near the point V2 on a cutting plane and the *y* mirror plane, respectively. Similar to the case of Fig. 4A, the two C lines have opposite chirality but the same polarization index *I*_pl_ = −1/2, and both puncture inwardly through the mirror plane at the V2 point, which is consistent with the phase index *I*_ph_ = −2 around the V2 point as shown by Arg(Ψ) on the mirror plane.

It is known that interesting Möbius strips of polarizations appear around a single C line (*6*, *39*, *40*, *44*, *45*). In general, a polarization strip with an odd number of twists must be topologically nontrivial, while a stripe with an even number of twists can always be deformed to a cylinder with trivially aligned polarizations (*25*). An interesting question is: Could mirror symmetry enrich the topological classification and give rise to nontrivial polarization structures around the nexus of C lines? To explore this question, let us consider the polarization major axes **A** on a transverse self-mirror symmetric (TSMS) loop (i.e., a loop that intersects with and is symmetric about the *y* mirror plane). Since the two mirror-partner C lines have opposite directions, the phase index for this loop vanishes, which seems to indicate a trivial topology on the loop with boring spatial structure of **A**. However, under the constraint of mirror symmetry, the topological classification along such TSMS loops is determined by the relative homotopy group π_1_(*X*, *X*_Π_) = π_1_((*S*^2^ × *S*^1^)/ℤ_2_, *S*^1^) = ℤ_2_ (see the Supplementary Materials). Consequently, despite carrying zero phase index, the TSMS loops can still have a nontrivial ℤ_2_ topology manifesting as a mirror-symmetric double-twist Möbius strip of the polarization major axes **A**. Concretely, the major axis **A** on the mirror plane must point in the $\pm \hat{y}$ direction due to the *y*-mirror symmetry. The evolution of **A** on the TSMS loops gives rise to only two possible cases: trivial and nontrivial topological loops characterized by parallel and antiparallel major axes **A** at the two points piercing the mirror plane, respectively. Along a trivial loop, the polarizations can be continuously deformed into an untwisted strip. In contrast, the polarizations along a nontrivial loop must be twisted once at each side of the mirror plane and hence form a mirror-symmetric double-twist Möbius strip protected by mirror symmetry. We note that the absolute times of winding of 3D polarizations are not a topological invariant, which can change arbitrary even number of times by deforming either the loop or the system without breaking the symmetry (*25*, *39*). Thus, here, the number of twists should be counted in a topologically stable sense (i.e., the minimal number of twists that cannot be untied by continuous deformation). In addition, a mirror symmetry–protected double-twist (trivial) polarization strip must enclose an odd (even) number of C lines at each side of the mirror plane (see the Supplementary Materials). The above analysis is confirmed by the numerical results in Fig. 4 (A and B), where the pink arrows denote the polarization major axes **A** on TSMS loops around the V1 and V2 points, respectively. We notice the mirror-symmetric double-twist Möbius strips in both cases. This also explains why the two oppositely directed C lines at the two sides of the mirror plane cannot annihilate but must form a V point when meeting on the mirror plane. Once the mirror symmetry is broken, the double-twist Möbius strip can be deformed into a trivial strip with no twist, and the two C lines will be gapped at the V point. Therefore, our study reveals that spatial symmetry can engender intriguing topologically nontrivial polarization configurations that cannot stably exist in the nonsymmetric case.

### Integration of topology and generalized rotational symmetry

Higher-order PSs/PSLs are generally unstable without symmetry protection and can easily transform into multiple lowest-order PSs/PSLs under perturbations (*46*). The V points in Fig. 4 are a type of higher-order PSs protected by mirror symmetry. It is interesting to explore whether higher-order C points/lines can appear if the topology of structures is integrated with rotational symmetry.

**Fig. 5.**
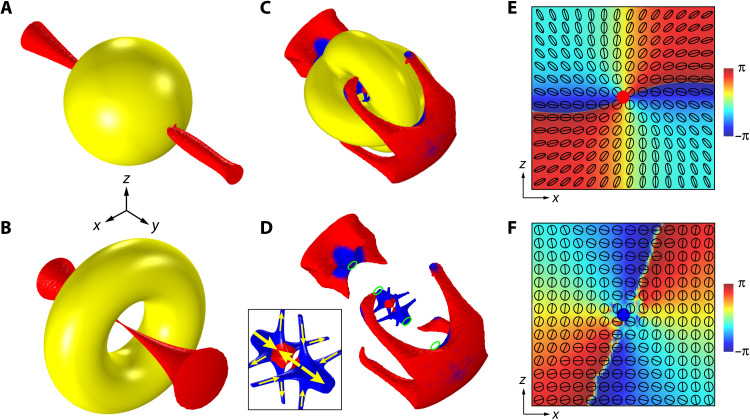
Higher-order C lines due to generalized rotational symmetry. (**A** and **B**) Higher-order C lines with index *I*_pl_ = +1 generated by a sphere and a torus, respectively. (**C** and **D**) Higher-order C lines generated by a torus nexus with *C*_4_ rotational symmetry. The torus nexus is removed in (D) to enable a clear view of the internal structures of the C lines. The green circles mark the four C points with *I*_pl_ = −1 on the surface of the torus nexus. The other C points off the center axis has index *I*_pl_ = −1/2 (blue) and *I*_pl_ = +1/2 (red). The inset shows a zoom-in of the center C lines, where the yellow arrows show the directions of the C lines. (**E** and **F**) Polarization ellipses and Arg(Ψ) for the C point at the center of the torus nexus and the C point marked by the leftmost green circle in (D), respectively.

We consider the metal sphere under the excitation of a right-handed circularly polarized plane wave $H_{\text{in}}=H_{0}(\hat{z}+i\hat{x})e^{\mathrm{iky}}$ that transforms under rotation about the *y* axis as *R*(ϕ)**H**_in_(*R*(−ϕ)**r**) = *e*^−*i*ϕ^**H**_in_(**r**) (*47*, *48*), where *R*(ϕ) denotes the rotation matrix with rotation angle ϕ. The rotation matrix *R*(ϕ) has three eigenvalues *e*^−*i*ϕ^, *e*^*i*ϕ^ and 1 with the corresponding eigenvectors $R=(\hat{z}+i\hat{x})/\sqrt{2}$, $L=(\hat{z}-i\hat{x})/\sqrt{2}$, and $\hat{y}$. Since the structure is axially symmetric about the *y* axis, the total magnetic field should be invariant against continuous rotation about the *y* axis up to a certain additional phase *R*(ϕ)**H**(*R*(−ϕ)**r**) = *e*^−*i*ϕ^**H**(**r**), which we dubbed as the generalized cylindrical symmetry ${\bar{C}}_{\infty }$ to differentiate it from the ordinary rotational symmetry (i.e., the system returns to itself without the additional phase after rotation). Along the *y* axis, the magnetic field satisfies $R(\phi )H(y\hat{y})=e^{-i\phi }H(y\hat{y})$, implying that **H** is the circularly polarized eigenvector of *R*(ϕ) with the same chirality as the incident field, i.e., $H\propto R=(\hat{z}+i\hat{x})/\sqrt{2}$; hence, the magnetic spin **S** is fixed in the $+\hat{y}$ direction. Moreover, since the scalar field satisfies Ψ(*R*(ϕ)**r**) = *e*^2*i*ϕ^Ψ(**r**), the phase index around the *y* axis is quantized as *I*_ph_ = 1/(2π) ∮ *d*ϕ∂_ϕ_Arg(Ψ(*R*(ϕ)**r**)) = +2, and accordingly, $I_{\text{pl}}=\text{sign}(S\cdot \hat{y})I_{\text{ph}}/2=+1$, indicating that the total field along the *y* axis forms a second-order C line. This prediction is confirmed by the numerical result in Fig. 5A, where the red line threading the sphere corresponds to a C line with index *I*_pl_ = +1, and the index sum on the surface of the sphere remains unchanged, i.e., ∑*I*_pl_ = +1 × 2 = +2. Similar phenomenon also happens in the single torus case, as shown in Fig. 5B. However, the vanished Euler characteristic (χ = 0) of the torus makes the C line with *I*_pl_ = +1 thread the hole, without touching the metal surface. It is worth noting that when the incident field is left-handed plarized **H**_in_ ∝ **L**, *I*_ph_ and **S** will reverse their signs simultaneously, while the polarization index of the C line will be left intact, i.e., *I*_pl_ = +1, as the result of the only possible configuration of a cylindrically symmetric line field vortex (see fig. S4).

When the structure illuminated by the circularly polarized wave **H**_in_ has a discrete rotational symmetry *C_n_* about the *y* axis, the total magnetic field respects the generalized rotational symmetry ${\bar{C}}_{n}$: *R*(2π/*n*)**H**(*R*(−2π/*n*)**r**) = *e*^−*i*2π/*n*^**H**(**r**). The discrete ${\bar{C}}_{n}$ symmetry can also protect that the total field forms a C line with **H** ∝ **R** along the *y* axis when *n* ≥ 3 (for *n* = 1,2, since the two eigenvalues *e*^*i*2π/*n*^, *e*^−*i*2π/*n*^ of *R*(2π/*n*) are degenerate, **H** can be arbitrarily polarized along the *y* axis, and there is no C line on the *y* axis), and the order of the C line depends on the order *n* of the rotational symmetry. Concretely, since the ${\bar{C}}_{n}$ symmetric scalar field satisfies Ψ(*R*(2π/*n*)**r**) = *e*^*i*4π/*n*^Ψ(**r**), the phase index along a circle, *c*, symmetric about the *y* axis is *nℤ* quantized: *I*_ph_(*c*) = 2 + *nm* (see the Supplementary Materials for a rigorous proof), where *m* is an arbitrary integer. The first term of *I*_ph_(*c*) (i.e., constant 2) corresponds to the phase index of systems respecting the generalized cylindrical symmetry ${\bar{C}}_{\infty }$ and hence must also obey all discrete ${\bar{C}}_{n}$ symmetries. The second term is due to the fact that the ${\bar{C}}_{n}$ symmetry ensures that the C lines must enter or exit the circle in groups, each with *n* copies *C_n_*-symmetrically aligned around the *y* axis. Therefore, the polarization and phase indices of the central C line are determined by the minimal value of the phase index of the circle around the *y* axis: $I_{\text{pl}}=+I_{\text{ph}}^{\text{min}}/2$ with $I_{\text{ph}}^{\text{min}}\in \{I_{\text{ph}}(c)||I_{\text{ph}}(c)|=\text{min}|2+\text{nm}|,m\in Z\}$. For *n* = 1,2, the minimal charge is $I_{\text{ph}}^{\text{min}}=0$, which is consistent with our prediction that there is no C line on the *y* axis; for *n* = 3, the central C line is first order with $I_{\text{pl}}=I_{\text{ph}}^{\text{min}}/2=-1/2$; for *n* = 4, the central C line is second order with either *I*_pl_ = +1 or *I*_pl_ = −1; for *n* ≥ 5, the C line must be second order with positive index *I*_pl_ = +1 (see the Supplementary Materials for an alternative proof via perturbation analysis). Akin to the cylindrical symmetry case, a left-handed circularly polarized incident wave illuminating a *C_n_* symmetric structure will induce opposite $I_{\text{ph}}^{\text{min}}$ and **S** but an identical *I*_pl_, in comparison to those generated by a right-handed circularly polarized incident wave. This can also be understood from the compatibleness between the line field configurations and the rotational symmetries.

As the most interesting case, we generate the central C lines with *I*_pl_ = ±1 protected by the ${\bar{C}}_{4}$ rotational symmetry, using the torus nexus with genus *g* = 3 in Fig. 5C. Under the incidence of the same circularly polarized plane wave, this structure generates complexly distributed C lines. For the ease of visibility, we show the C lines without the torus nexus in Fig. 5D. A total of 20 C points appear on the surface of the torus nexus: 4 points with index *I*_pl_ = −1 (marked by the green circles), 8 points with index *I*_pl_ = −1/2 (corresponding to the tails of the blue lines without a green circle), and 8 points with index *I*_pl_ = +1/2 (corresponding to the tails of the red lines). The index sum is ∑*I*_pl_ = −4 and agrees with the structure’s Euler characteristic χ= 2 − 2 × 3 = −4 Figure 5E shows the polarization ellipses and Arg(Ψ) for the center C point on the *y* mirror plane, where an index *I*_ph_ = 2*I*_pl_ = +2 is clearly observed (**t**_c_ points in $+\hat{y}$ direction). Figure 5F shows the polarization ellipses and Arg(Ψ) for the C point marked by the leftmost green circle in Fig. 5D, which clearly shows an index *I*_ph_ = 2*I*_pl_ = −2.

In the center region of the C lines, as shown by the inset in Fig. 5D, there are two transition points along *y* axis where the polarization index changes as *I*_pl_ = −1 → +1 → −1. At the transition points, four additional first-order C lines (blue) grow out in transverse directions, forming a nexus of C lines. The directions **t**_c_ of the C lines are denoted by the yellow arrows. This remarkable structure of C lines arises from the conservation of the phase index *I*_ph_. Since ${\bar{C}}_{4}$ symmetry supports two different polarization indices *I*_pl_ = ±1 of the central C line, the phase index defined on a loop encircling the central C line should be *I*_ph_ = 2*I*_pl_ = ±2, and the sign of the index determines the direction of the C line. When moving the loop along the central C line, the conservation of the phase index forbids the loop to pass through the transition point of two oppositely directed C-line segments (labeled by the large yellow arrows along *y* axis) without crossing other PSLs, which implies the emergence of the other four transverse C lines connecting with the transition point due to the ${\bar{C}}_{4}$ symmetry. The existence of these “budding” C lines is also confirmed via the perturbation analysis near the *y* axis (see the Supplementary Materials). Still, by the conservation of *I*_ph_ and ${\bar{C}}_{4}$ symmetry, the four budding C lines are first order and must be directed either all outward or all inward such that the total “arrows” weighted by ∣*I*_ph_∣ flowing to each nexus point are equal to zero, as shown by the smaller yellow arrows in the inset of Fig. 5D.

## DISCUSSION

PSs can emerge in various parameter spaces. In momentum space, they are determined by the Bloch states and are intimately related to the BICs in periodic photonic structures, where V points can give rise to vanishing far-field radiation of Bloch states (*49*, *50*). PSs also provide a direct way to visualize the band Chern number and the topological structure of EPs (*32*, *51*). Such studies typically focus on the PSs that live on the Brillouin torus associated with a 2D periodic structure. Extension to other parameters spaces of different topology (genus) is difficult, if not impossible. In the real space, the PSs are attributed to the interference of the Fourier plane waves of the incident and scattering fields. This endows the real-space PSs with several unique properties. They can live on various 2D manifolds and give rise to intriguing PSLs whose configurations can be easily controlled via the topology and symmetry of optical structures, as we have demonstrated here. In addition, the PSs in 3D space naturally carry Pancharatnam-Berry phase and spin-redirection phase (*25*), offering rich mechanisms for manipulating light’s polarization and phase. Because of their intrinsic chirality deriving from spin and inhomogeneous phase distribution in the near field, the emergence of the PSs is usually accompanied by the spin-orbit interactions of light (*52*). Such spin-orbit interactions can enable directional near-field coupling and far-field radiation with fruitful applications in designing optical sources and nanoantennas (*53*). Experimentally probing these PSLs can be achieved by using the near-field characterization techniques that can map subwavelength longitudinal and transverse field components, such as scanning near-field optical microscopy (*54*).

In sum, we establish a direct relationship between the topology of metal structures and the magnetic PSLs in the near field. We show that the index sum of the PSs born on the surface of the structures is solely determined by the structures’ Euler characteristic due to the tangent nature of magnetic field near the metal surface. In addition, we find that the interplay of topology with mirror symmetry or generalized rotational symmetry can give rise to topologically stable higher-order PSs/PSLs with richer morphologies including C line nexuses and mirror-symmetric double-twist Möbius strip of polarizations. The topological properties of the PSLs can be well understood by using a non-Hermitian two-band Hamiltonian, where the topological classification of non-Hermitian EP lines can be mapped to that of C lines characterized by the phase index. According to this correspondence, the merging, bifurcation, and topological transition of C lines in the real space must observe the phase index conservation law. It is worth emphasizing that the birth of PSs in the structures is protected by the Poincaré-Hopf theorem that has nothing to do with the type of the incident wave, as long as the fields concerned are tangent. Excitations other than plane waves can only change the local distributions of those PSs, but their global properties are fully source independent: The index sum of the PSs on the surface must be equal to the Euler characteristic of the structures (see the Supplementary Materials for the examples of the PSs generated by incident circularly polarized vector Bessel beams). These conclusions apply to frequency regimes with small skin depths of the metals. For metals at other frequencies and dielectric materials with deeper penetration depths, neither total electric nor magnetic fields are tangent, and consequently, the existence of PSs is not protected on the surface. However, this limitation by no means implies that our study is of little practical value. For many near-field applications involving 2D materials, the interactions involve only tangent fields, for which our theoretical analyses can be directly applied. Moreover, for many optical resonators, only one set of modes can be excited by some sources, with either the electric or magnetic fields being purely tangent (e.g., TE and TM modes in Mie theory for spherical particles), where our theory can play a substantial role.

Our study uncovers exotic topological properties of optical polarization fields that are irrelevant to the specific material or geometry of the optical structures. The results have essentially connected PSs, the topology and symmetry of structures, as well as non-Hermitian physics, opening extra opportunities for fundamental conceptual explorations and many related practical applications in chiral discrimination and sensing, chiral quantum optics, and topological photonics. They may also be extended to other classical wave systems such as sound waves and water surface waves (*55*, *56*).

## MATERIALS AND METHODS

### Homotopy approach characterizing the topology of C lines

According to the approach for classifying the gapless topological phases of non-Hermitian Hamiltonians (*43*), the degenerate submanifold *X*_D_ (i.e., EPs and nondefective degeneracies) is regarded as the topological obstruction in the configuration space of the Hamiltonians $X_{H}=\{H=H\cdot \vec{\sigma }:H\in {\mathbb{C}}^{3}\}$, the existence of which induces the nontrivial connectivity to the subspace of *X_ℋ_* excluding the degeneracies *X* = *X_ℋ_* − *X_D_* = {ℋ ∈ *X_ℋ_* : **H** · **H** ≠ 0} (i.e., the subspace with separable bands). Tracing a closed loop in *X*, if the loop is noncontractible via continuous deformation, then it must encircle the degenerate submanifold in an inextricable manner. Therefore, the topology of the EP lines is essentially encoded by the topological classification of the loops in the nondegenerate subspace *X*, mathematically given by the first homotopy group of *X*. Homotopy refers to a topological equivalence relation. For two loops in *X* that can be continuously transformed to each other, they belong to the same homotopy equivalence class and hence characterize the same topological phases of degeneracies. In addition, the first homotopy group is just the group formed by all homotopy equivalence classes of loops in *X*. The configuration space of the non-Hermitian Hamiltonian without degeneracies is given by $X=(\frac{GL_{2}(\mathbb{C})}{GL_{1}(\mathbb{C})\times GL_{1}(\mathbb{C})}\times \text{Con}f_{2}(\mathbb{C}))/{\mathbb{Z}}_{2}$ (*43*), where the first part formed by the coset space of the complex general linear groups *GL_n_*(ℂ) represents the space spanned by the two eigenstates of the Hamiltonian, the second part Conf_2_(ℂ) = {{*h*_+_, *h*_−_} ∈ ℂ : *h*_+_ ≠ *h*_−_} denotes the configuration space of two separable complex eigenvalues of ℋ(**r**), and the divisor ℤ_2_ denotes the redundancy that the eigenstates and eigenvalues change the orders simultaneously. Moreover, it is shown that the eigenstate and the eigenvalue parts are homotopy equivalent to a sphere and a circle, i.e., $\frac{GL_{2}(\mathbb{C})}{GL_{1}(\mathbb{C})\times GL_{1}(\mathbb{C})}≃S^{2}$ and Conf_2_(ℂ) ≃ *S*^1^, respectively (*43*). Therefore, the configuration space for topological classification can be rewritten as *X* ≃ (*S*^2^ × *S*^1^)/ℤ_2_, which can be physically interpreted as the space of the magnetic polarizations (see the section “Mapping to non-Hermitian EPs”).

### Numerical simulations

Full-wave numerical simulations were performed by using a finite-element package COMSOL Multiphysics. The considered structures are made of gold characterized by a Drude model $\varepsilon_{\text{Au}}=1-\omega_{p}^{2}/(\omega^{2}+i\omega \omega_{t})$, where ω_p_ = 1.28 × 10^16^ rad/s and ω_t_ = 7.10 × 10^13^ rad/s (*57*). The spheres have radii of 500 nm. The inner and outer radii of the single torus in Fig. 1C is *r* = 500 nm and *R* = 1500 nm. For the double torus in Fig. 1D, we set *r* = 250 nm and *R* = 750 nm, and the center distance between the holes is 500 nm. In all cases of Fig. 1, we assume a plane wave propagating in *z* direction, and the magnetic field is linearly polarized in *y* direction, and open boundary conditions are applied. In the cases of Fig. 5, we assume that the incident plane wave propagates in *y* direction and is circularly polarized.

## Supplementary Materials

### This PDF file includes:

Supplementary Text Figs. S1 to S6 References
