# Supplementary material for: Topological near fields generated by topological structures
Source: Sci Adv. 2022 Oct 14;8(41):eabq0910. doi: 10.1126/sciadv.abq0910 (PMC9565808; doi:10.1126/sciadv.abq0910)
Supplement: Supplementary file 1 — Supplementary Text Figs. S1 to S6 References [file sciadv.abq0910_sm.pdf]

Science Advances
AAAS

Supplementary Materials for

Topological near fields generated by topological structures

Jie Peng *et al.*

Corresponding author: Shubo Wang, shubwang@cityu.edu.hk

Sci. Adv. **8**, eabq0910 (2022)
DOI: 10.1126/sciadv.abq0910

This PDF file includes:

Supplementary Text
Figs. S1 to S6
References

Supplementary Text

Topological mapping of the near field

Under the excitation of an incident wave, currents will be induced in the metal structures. If the skin depth is much smaller compared to the dimension of the structures, the induced currents localize on a thin surface layer. In this case, the magnetic field **H** near the surface is dominated by the tangent component $H_{\parallel}$. Figure S1 shows the numerical simulation results of $H_{\perp}/|\mathbf{H}|$ for structures with different genus (i.e., single sphere, double spheres, torus, double-torus, and torus nexus) and within the frequency range of interest. We see that the normal components $H_{\perp}$ of the magnetic field are indeed very small. Thus, the corresponding polarization ellipses of the magnetic field are parallel to the surface. The polarization major axis **A** can be mapped to a line field defined on a 2D smooth manifold (i.e., the surface of the structures). Applying the Poincaré-Hopf theorem to this line field, we then can predict the total topological index of the singularities (i.e., C points and V points).

Polarization index and phase index

In previous studies, two different ways to define the topological indices of a C line in 3D space, i.e., the definitions with the polarization ellipse and with the phase winding number, were proposed and their intrinsic relation had been explored (*13, 25, 41*). Here, we review these two kinds of topological indices from the perspective of topological classification.

1. Local and global polarization indices

As shown in Fig. S2A, consider a C point, $\mathbf{r}_\mathrm{c}$, with circular polarization $\mathbf{H}_\mathrm{c} = H_c(\mathbf{e}_1 + \mathrm{i}\mathbf{e}_2)/\sqrt{2}$, where $\mathbf{e}_1$ and $\mathbf{e}_2$ are orthogonal unit vectors in the local polarization plane of $\mathbf{H}_\mathrm{c}$. Near the C point, the magnetic spins are approximately aligned along the same direction $\mathbf{S}_\mathrm{c} = \mathbf{S}(\mathbf{r}_\mathrm{c}) = 2{H_\mathrm{c}}^2\, \mathbf{e}_1 \times \mathbf{e}_2$, namely the polarization ellipses are approximately in the same plane perpendicular to $\mathbf{e}_\mathrm{s} = \mathbf{S}_\mathrm{c}/(2H_\mathrm{c}^2)$, characterized by the major axis $\mathbf{A} = A_1\mathbf{e}_1 + A_2\mathbf{e}_2 + O(\mathbf{r} - \mathbf{r}_\mathrm{c})\mathbf{e}_\mathrm{s}$. As such, we can assign a local polarization index to the C point as the winding number of the major axis in the local polarization plane

$$
\begin{aligned}
I_\mathrm{pl}(\mathbf{r}_\mathrm{c}) = & \lim_{\gamma\to\mathbf{r}_\mathrm{c}} \frac{1}{2\pi}\oint_\gamma d\arctan\left(\frac{A_2}{A_1}\right) = \lim_{\gamma\to\mathbf{r}_\mathrm{c}} \frac{1}{2\pi}\oint_\gamma \frac{1}{A^2}(\mathbf{A}\times d\mathbf{A})\cdot\mathbf{e}_\mathrm{s} \\
= & \lim_{\gamma\to\mathbf{r}_\mathrm{c}} \frac{1}{2\pi}\oint_\gamma \frac{1}{AB}(\mathbf{B}\cdot d\mathbf{A}) = \lim_{\gamma\to\mathbf{r}_\mathrm{c}} \frac{1}{2\pi}\oint_\gamma \frac{2}{{H_\mathrm{c}}^2}(\mathbf{B}\cdot d\mathbf{A})
\end{aligned}
\quad \text{(S1)}
$$

where the integration is along an infinitesimal circle γ around $\mathbf{r}_\mathrm{c}$ with the positive normal vector $\mathbf{S}_\mathrm{c}$ (defined with respect to the right-hand rule of γ). If we map the transverse polarization ellipses near $\mathbf{r}_\mathrm{c}$ to the Poincaré sphere, the azimuthal angle on the sphere is given by $\varphi = 2\arctan(A_2/A_1)$. Thus, the local polarization index can be equivalently expressed as $I_\mathrm{pl} = \lim_{\gamma\to\mathbf{r}_\mathrm{c}} \frac{1}{4\pi}\oint_\gamma d\varphi$. We note that all the polarization indices mentioned in the main text refer to this local polarization index, unless otherwise stated. Equation (S1) shows that I_pl is just given by the geometric phase integrated by the Euler connection (differential 1-form) $a_\mathrm{Eu} = \frac{1}{AB}(\mathbf{B}\cdot d\mathbf{A}) = \mathbf{e}_\mathrm{B}\cdot d\mathbf{e}_\mathrm{A}$ of the orthonormal frame $(\mathbf{e}_\mathrm{A}, \mathbf{e}_\mathrm{B}) = \left(\frac{\mathbf{A}}{A}, \frac{\mathbf{B}}{B}\right)$. And $\lim_{\mathbf{r}\to\mathbf{r}_\mathrm{c}} A = \lim_{\mathbf{r}\to\mathbf{r}_\mathrm{c}} B = H_\mathrm{c}/\sqrt{2}$ has been used in the last step. In the absence of symmetry protection, only first order C lines can stably exist in the space, around which the major axes form a half vortex, hence the local polarization index of a first-order C line is given by $I_\mathrm{pl} = \pm 1/2$. However, there may exit some singular points on a C line at

which $\mathbf{S}_\mathrm{c}\cdot\mathbf{t}=0$, and one cannot find a circle γ that is both normal to $\mathbf{S}_\mathrm{c}$ and encircles the C line at such a singular point. As a result, the sign of I_pl can flip at such points, and hence the local polarization index I_pl is not a conserved quantity along a C line. Such sign flips of I_pl are very common and occur in almost all the cases considered in the main text. As an additional note, the local polarization index of a V point in 3D polarization fields is indeed ill-defined, since in general there does not exist a special direction, as the direction of $\mathbf{S}$ for a C point, that tends to be perpendicular to all polarization ellipses near the V point. Nevertheless, the *ad hoc* polarization indices of the accidental V lines shown in Fig. 1 is suitable for the application of Poincaré-Hopf index theorem. This is because the magnetic fields are perpendicular to the surface normal on the structural surfaces which forms a fixed rotation axis of the tangent polarization ellipses around the V point on the surface.

On the other hand, the definition of the local polarization index in Eq. (S1) cannot be used to define the index of 3D polarizations along a loop with finite size, since the integral $\oint a_\mathrm{Eu}$ is not quantized in general. If we only concern about the major axis $\mathbf{A}$ of the polarization ellipse, the globally conserved polarization index along an arbitrary loop, γ, can be introduced by the homotopy equivalence class of loops in the real projective space $\mathbb{R}P^2$ (the space of $\mathbf{A}$) (*58*):

$$\nu_\mathrm{pl}(\gamma)=0 \text{ or } 1\in\pi_1(\mathbb{R}P^2)=\mathbb{Z}_2 \tag{S2}$$

This shows that the alignments of the major axis along a loop γ can only be classified into two topological phases (homotopy classes), i.e., the trivial phase ($\nu_\mathrm{pl}(\gamma)=0$) corresponding to the untwisted alignment of the major axes and the nontrivial phase ($\nu_\mathrm{pl}(\gamma)=1$) corresponding to the Möbius twisted alignment (see Fig. S2B). Note that here untwisted alignment and Möbius twisted alignment should be understood in a topologically stable sense. Although Multi-twisted Möbius strips of polarization had been widely studied in literature, the twist number n (≥ 2) is not a topological invariant but can change under the continuous deformation of the loop or of the optical fields (this is because the polarization ellipses are not solid stuffs, and the self-intersection of the polarization strip is allowed). Only the parity of the twist number, i.e., $\nu_\mathrm{pl}(\gamma)$, is topologically stable against the continuous deformation (*25*).

2. Phase index

A general monochromatic magnetic field can be expressed as $\mathbf{H}=(\mathbf{A}+i\mathbf{B})e^{\mathrm{i}\theta}$, where $\theta=\mathrm{Arg}\,(\mathbf{H}\cdot\mathbf{H})/2$ is a proper phase. As we have shown in the main text, by introducing the scalar field $\Psi=\mathbf{H}\cdot\mathbf{H}=H^2e^{\mathrm{i}2\theta}$, the phase winding number of the scalar field Ψ along a loop γ, $I_\mathrm{ph}(\gamma)=\frac{1}{2\pi}\oint_\gamma d\,\mathrm{Arg}(\Psi)=\frac{1}{\pi}\oint_\gamma d\,\theta\in\mathbb{Z}$, gives a complete topological classification of the C lines enclosed by 1D loops (*43*). Here, we consider the relation between the phase index I_ph, the global polarization index ν_pl, and the local polarization index I_pl.

First, we consider a loop γ with finite size. Starting at a base point $\mathbf{r}_0$ on the loop, we examine the continuous evolution of the major-axis bivector and the phase, i.e., $(\mathbf{A},e^{\mathrm{i}\theta})$, along the loop, which begins as $(\mathbf{A}_0,e^{\mathrm{i}\theta_0})$ at the base point $\mathbf{r}_0$ and finally turns to $(\mathbf{A}_\mathrm{f},e^{\mathrm{i}\theta_\mathrm{f}})$ when returning to $\mathbf{r}_0$. Since $\mathbf{A}e^{\mathrm{i}\theta}(\mathbf{r}_0)=\mathbf{A}_0e^{\mathrm{i}\theta_0}=\mathbf{A}_\mathrm{f}e^{\mathrm{i}\theta_\mathrm{f}}$, we have either $(\mathbf{A}_\mathrm{f},e^{\mathrm{i}\theta_\mathrm{f}})=(\mathbf{A}_0,e^{\mathrm{i}\theta_0})$ or $(\mathbf{A}_\mathrm{f},e^{\mathrm{i}\theta_\mathrm{f}})=(-\mathbf{A}_0,-e^{\mathrm{i}\theta_0})$. Thus, we obtain the relation between the phase index and the global polarization index along the loop γ:

$$\exp[\mathrm{i}\pi I_\mathrm{ph}(\gamma)]=\exp\left[\mathrm{i}\oint_\gamma d\theta\right]=\exp[\mathrm{i}(\theta_\mathrm{f}-\theta_0)]=\mathrm{sign}(\mathbf{A}_0\cdot\mathbf{A}_\mathrm{f})=\exp[\mathrm{i}\pi\nu_\mathrm{pl}(\gamma)] \tag{S3}$$

or equivalently,

$$\nu_{\mathrm{pl}}(\gamma) = I_{\mathrm{ph}}(\gamma) \bmod 2 \tag{S4}$$

As a result, we know that if the major axes form a trivial untwisted (Möbius twisted) strip along a loop, the loop encloses even (odd) numbers of first-order C lines. Hence, although both the phase index $I_{\mathrm{ph}}(\gamma)$ and the global polarization index $\nu_{\mathrm{pl}}(\gamma)$ along a loop are invariant against continuous deformations, only the phase index can distinguish all inequivalent topological phases associated with C lines enclosed by the loop.

In contrast to the phase index $I_{\mathrm{ph}}(\gamma)$ and the global polarization index $\nu_{\mathrm{pl}}(\gamma)$ that are generally defined for a loop γ in the 3D space, the local polarization index $I_{\mathrm{pl}}(\mathbf{r}_{\mathrm{c}})$ characterizes the property of a certain C point $\mathbf{r}_{\mathrm{c}}$. Even so, if a loop $\gamma_{\mathbf{t}}$ only encircles a single C line as shown in Fig. S2A, the corresponding phase index $I_{\mathrm{ph}}(\gamma_{\mathbf{t}})$ serves as a minimal charge of the C line and has a definite relation to the local polarization index I_{pl} at each point on the C line. To derive this relation, we introduce the complex polarization vector $\mathbf{C} = \mathbf{A} + \mathrm{i}\mathbf{B} = \mathbf{H}e^{-i\theta}$, the Berry connection of the complex vector $\mathbf{C}$ is identical with the Euler connection of the real vector frame

$$a_{\mathrm{Berry}}(\mathbf{C}) = \frac{\mathrm{i}\mathbf{C}^* \cdot d\mathbf{C}}{|C|^2} = \frac{\mathbf{B} \cdot d\mathbf{A} - \mathbf{A} \cdot d\mathbf{B}}{H^2} = \frac{2}{H^2}(\mathbf{B} \cdot d\mathbf{A}) \tag{S5}$$

where $d(\mathbf{B} \cdot \mathbf{A}) = \mathbf{B} \cdot d\mathbf{A} + \mathbf{A} \cdot d\mathbf{B} = 0$ has been used. Therefore, the local polarization index of a C point $\mathbf{r}_{\mathrm{c}}$ can be expressed as

$$I_{\mathrm{pl}}(\mathbf{r}_{\mathrm{c}}) = \lim_{\gamma_{\mathbf{t}} \to \mathbf{r}_{\mathbf{c}}} \mathrm{sign}(\mathbf{S}_{\mathrm{c}} \cdot \mathbf{t}) \frac{1}{2\pi} \oint_{\gamma_{\mathbf{t}}} a_{\mathrm{Eu}} = \lim_{\gamma_{\mathbf{t}} \to \mathbf{r}_{\mathbf{c}}} \mathrm{sign}(\mathbf{S}_{\mathrm{c}} \cdot \mathbf{t}) \frac{1}{2\pi} \oint_{\gamma_{\mathbf{t}}} a_{\mathrm{Berry}}(\mathbf{C}) \tag{S6}$$

where $\mathbf{t}$ is a tangent vector of the C line with the direction complying with the right-hand rule of the loop $\gamma_{\mathbf{t}}$. Further, according to $\mathbf{C} = \mathbf{H}e^{-i\theta}$, we have

$$a_{\mathrm{Berry}}(\mathbf{C}) = \frac{\mathrm{i}\mathbf{C}^* \cdot d\mathbf{C}}{|C|^2} = \frac{\mathrm{i}\mathbf{H}^* \cdot d\mathbf{H}}{|H|^2} + d\theta = a_{\mathrm{Berry}}(\mathbf{H}) + \frac{1}{2} d\mathrm{Arg}(\Psi) \tag{S7}$$

Since $\mathbf{H}$ field is continuous at $\mathbf{r}_{\mathrm{c}}$ with no singularity, the integration of $a_{\mathrm{Berry}}(\mathbf{H})$ along the infinitesimal circle must vanish, and hence we can obtain the relation between the local polarization index of a C point and the phase index along an infinitesimal circle $\gamma_{\mathbf{t}}$ around the C point, provided that $\mathbf{S}_{\mathrm{c}} \cdot \mathbf{t} \neq 0$ (*41*):

$$\begin{aligned} I_{\mathrm{pl}}(\mathbf{r}_{\mathrm{c}}) &= \mathrm{sign}(\mathbf{S}_{\mathrm{c}} \cdot \mathbf{t}) \left[\lim_{\gamma_{\mathbf{t}} \to \mathbf{r}_{\mathbf{c}}} \frac{1}{2\pi} \oint_{\gamma_{\mathbf{t}}} a_{\mathrm{Berry}}(\mathbf{H}) + \frac{1}{4\pi} \oint_{\gamma_{\mathbf{t}}} d\,\mathrm{Arg}(\Psi) \right] \\ &= \mathrm{sign}(\mathbf{S}_{\mathrm{c}} \cdot \mathbf{t}) \frac{1}{4\pi} \oint_{\gamma_{\mathbf{t}}} d\,\mathrm{Arg}(\Psi) = \frac{1}{2} \mathrm{sign}(\mathbf{S}_{\mathrm{c}} \cdot \mathbf{t}) I_{\mathrm{ph}}(\gamma_{\mathbf{t}}) \end{aligned} \tag{S8}$$

In terms of the phase index along the loop $\gamma_{\mathbf{t}}$ encircling the C line, we can uniquely define a positive direction to the C-line by the directed tangent vector (see Fig. S2A)

$$\mathbf{t}_{\mathrm{c}} = \mathrm{sign}\left(I_{\mathrm{ph}}(\gamma_{\mathbf{t}})\right) \mathbf{t} = \mathrm{sign}(I_{\mathrm{pl}}\, \mathbf{S}_{\mathrm{c}} \cdot \mathbf{t}) \mathbf{t} \tag{S9}$$

Mirror symmetry protected $\mathbb{Z}_2$ topology of polarization on mirror-symmetric loops

1. Topology along semi-loop terminated on mirror plane – relative homotopy approach

Since the system respects mirror symmetry (say, the *y*-mirror symmetry as shown in Fig. S3), the distributions of the magnetic fields on the two sides of the mirror plane Π are one-to-one correspondent. Hence, to characterize the topology along a self-mirror-symmetric loop c, one only need to investigate a semi-loop of c on either side of Π (see Fig. S3A). For such a semi-loop γ (which is equivalent to a 1D disk D^1) terminated at two points $\mathbf{r}_0$, $\mathbf{r}_t$ ($\in \Pi$) on the mirror-plane and

supposing $\mathbf{r}_0 \in \Pi$ is fixed, while $\mathbf{r}_t$ can move freely in Π, the magnetic field $\mathbf{H}$ along γ defines a map (see Fig. S3A) (*58*):

$$\mathbf{H} \circ \gamma: \begin{cases} D^1 & \to X \simeq \dfrac{S^2 \times S^1}{\mathbb{Z}_2} \\ \partial D^1 & \to X_\Pi \simeq S^1 \\ \partial D^1 \ni \mathbf{r}_0 & \to \mathbf{H}_0 \in X_\Pi \end{cases} \tag{S10}$$

The first line of the map means that the magnetic fields of all points on γ belong to the configuration space X; the second line means that the two terminal points of γ belong to $X_\Pi = \{\mathbf{H} = H_y\hat{\mathbf{y}}, H_y \neq 0\}$; the third line indicates that the magnetic field at the starting point of γ is fixed to the base point $\mathbf{H}_0 \in X_\Pi$. The topological equivalence of $\mathbf{H}(\mathbf{r})$ along two semi-loops γ_1, γ_2 requires that the distributions of the magnetic fields on the two paths can continuously deform to each other without encountering a singular point (i.e. a C point or V point), which mathematically means $\mathbf{H} \circ \gamma_1 \sim_{X_\Pi} \mathbf{H} \circ \gamma_2$ ($\sim_{X_\Pi}$ denotes the homotopy equivalence relative to X_Π), namely there exists a homotopy (i.e. a continuous deformation between $\mathbf{H} \circ \gamma_1$and $\mathbf{H} \circ \gamma_2$) $F: D^1 \times [0,1] \to X$ relative to X_Π such that:

$$F(\cdot\,,0) = \mathbf{H} \circ \gamma_1, \quad F(\cdot\,,1) = \mathbf{H} \circ \gamma_2, \quad F(\partial D^1, t) \subset X_\Pi, \quad F(\mathbf{r}_0, t) \equiv \mathbf{H}_0 \tag{S11}$$

where the first (second) equality denotes that the function $F(\cdot, t)$ is equal to the map along γ_1 (γ_2) at $t = 0$ ($t = 1$), the third and last equalities indicate that the values of F at the terminals of the semi-loop are restricted in X_Π and the base point is fixed at $\mathbf{H}_0$ during the deformation.

Then we know that the topological classification of the magnetic fields along a semi-loop γ (or a self-mirror-symmetric loop) is determined by the relative homotopy group

$$\pi_1(X, X_\Pi, \mathbf{r}_0) = \pi_1\left(\frac{S^2 \times S^1}{\mathbb{Z}_2}, S^1, \mathbf{r}_0\right) = \{\mathbf{H} \circ \gamma\}/\sim_{X_\Pi} \tag{S12}$$

Since $X_\Pi \simeq S^1$ is path connected, $\pi_1(X, X_\Pi, \mathbf{r}_0) = \pi_1(X, X_{\Pi_1}, \mathbf{r}_0')$ for any two different base points $\mathbf{r}_0$, $\mathbf{r}'_0 \in \Pi$, so we may simply express the relative homotopy group as $\pi_1(X, X_\Pi)$. To compute the relative homotopy group $\pi_1(X, X_\Pi)$, we consider the natural fibration of the configuration space X, which is defined by the projection map from the configuration space X to the space of the major axis $\mathbb{R}P^2$ (*43*, *58*):

$$p: X \simeq \frac{S^2 \times S^1}{\mathbb{Z}_2} \quad \to \quad \mathbb{R}P^2 = \{\boldsymbol{A} \in S^2 | \boldsymbol{A} \sim -\boldsymbol{A}\} \tag{S13}$$

such that $p(\mathbf{H}) = [\pm\boldsymbol{A}]$ gives the bivector of the major axis of the polarization ellipse for each point $\mathbf{H} \in X$, and the fiber at each point $[\pm\mathbf{A}] \in \mathbb{R}P^2$: $p^{-1}([\pm\boldsymbol{A}]) = \{\mathbf{H} = \boldsymbol{A}e^{i\phi} | \phi \in [0,2\pi]\} \simeq S^1 \simeq U(1)$. Therefore, the projection p: $X \to \mathbb{R}P^2$ defines a $U(1)$-principal bundle on the base space $\mathbb{R}P^2$ (i.e. the "polarization sphere" of the major axis, as shown in Fig. S3D where any two antipodal points on the sphere are identified as the same point in $\mathbb{R}P^2$).

According to Theorem 4.41 in Ref. (*58*) for a fiber bundle $p: E \to B$ that the n −homotopy group of the bundle E relative to the fiber F is isomorphic to the n −homotopy group of the base manifold by the induced map p_*: $\pi_n(E, F, x_0) \to \pi_n(B, b_0)$ with $x_0 \in p^{-1}(b_0)$, we obtain the topological classification on the semi-loops protected by y-mirror symmetry

$$\pi_1(X, X_{\Pi_1}) = \pi_1\left(\frac{S^2 \times S^1}{\mathbb{Z}_2}, S^1\right) \overset{p_*}{=} \pi_1(\mathbb{R}P^2) = \mathbb{Z}_2 \tag{S14}$$

Recalling that $\mathbb{R}P^2$ is just the space of the major axis, Eq. (S14) reveals that the topology along semi-loops terminated in the mirror plane Π is absolutely determined by the twist of the major axes along the semi-loops, which are classified into two phases characterized by the two kinds of

homotopic inequivalent trajectories on the $\mathbb{R}P^2$ sphere. Since the major axes at the two terminal points of a semi-loop is fixed along the normal direction of Π corresponding to the south and north poles of the $\mathbb{R}P^2$ sphere (see Fig. S3D), the trajectories of $\mathbf{A}$ on the $\mathbb{R}P^2$ sphere should either form arcs connecting the two poles (these arcs is already closed in $\mathbb{R}P^2$), or form loops that start and end at the same pole. For the arcs on the $\mathbb{R}P^2$ sphere, the major axes exhibit an inextricable odd twist along the semi-loops (see Fig. S3B), thereby deemed as topologically nontrivial phase. In contrast, since the loops on the $\mathbb{R}P^2$ sphere are contractible to a point, the major axes $\mathbf{A}$ along the semi-loop exhibit a trivial twist (see Fig. S3C) and can be continuously deformed into the configuration that all $\mathbf{A}$ are parallelly aligned. Equivalently, along a self-mirror-symmetric loop c (the corresponding semi-loop is γ), the major axes in the trivial and nontrivial phases form an untwisted strip and a mirror-symmetric double-twisted Möbius strip, respectively, which can be characterized by the global polarization index

$$\nu_{\mathrm{pl}}(c) = 2\nu_{\mathrm{pl}}(\gamma) = (0 \text{ or } 2) \bmod 4 \qquad (S15)$$

with $\nu_{\mathrm{pl}}(\gamma) = 0 \text{ or } 1 \in \pi_1(X, X_\Pi) = \mathbb{Z}_2$. Comparing with Eq. (S4), we know that if the y-mirror symmetry is broken, both the trivial and nontrivial phases in Eq. (S14) will reduce to the trivial phase. Therefore, the mirror symmetry is crucial to preventing the double-twist strip from unknotting.

2. Relation between mirror-symmetry protected $\mathbb{Z}_2$ topology and C lines

Now we discuss the relation of the twist of the polarization vectors along the semi-loops, and the number of the C lines at one side of the mirror plane enclosed by the semi-loops. First, we connect the two end points $\mathbf{r}_0, \mathbf{r}_1$ of the semi-loop γ with an arbitrary curve $s_1 \subset \Pi$, and hence obtain a closed loop $\gamma_1 = \gamma \circ s_1$ (see Fig. S3A). Severing the loop γ_1 at the base point $\mathbf{r}_0$, we lift the major-axis bivectors and phase $(\mathbf{A}, e^{i\theta}) \sim (-\mathbf{A}, -e^{i\theta}) \in \frac{S^2 \times S^1}{\mathbb{Z}_2}$ to $(\boldsymbol{A}, e^{i\theta}) \in S^2 \times S^1$ with fixing a definite direction of $\mathbf{A}$ and a definite value of θ at each point along the severed path γ'_1 such that they are continuous along the path except for the breakpoint $\mathbf{r}_0$ (see Fig. S3B). Along s_1, $\mathbf{A}(\mathbf{r} \in s)$ remains fixed to $\hat{\mathbf{y}}$ or $-\hat{\mathbf{y}}$, i.e., we have $\frac{\mathbf{A}(\mathbf{k}\in s)}{|\mathbf{A}(\mathbf{k}\in s)|} \equiv \frac{\mathbf{A}_1}{|\mathbf{A}_1|}$ with $\mathbf{A}_1 = \mathbf{A}(\mathbf{r}_1)$. On the other hand, $\mathbf{A}e^{i\theta}$ is globally single-valued, $\mathbf{A}(\mathbf{r}_0)e^{i\theta(\mathbf{r}_0)} = \mathbf{A}_0 e^{i\theta_0} = \mathbf{A}_\mathrm{f} e^{i\theta_\mathrm{f}}$ where $(\mathbf{A}_0, e^{i\theta_0})$ and $(\mathbf{A}_\mathrm{f}, e^{i\theta_\mathrm{f}})$ denote the lift vectors and phases at the starting and end points of γ'_1(see Fig. S3B). These two facts lead to the equality

$$\exp[i\pi I_{\mathrm{ph}}(\gamma_1)] = \exp[i(\theta_\mathrm{f} - \theta_0)] = \mathrm{sign}(\mathbf{A}_0 \cdot \mathbf{A}_\mathrm{f}) = \mathrm{sign}(\mathbf{A}_0 \cdot \mathbf{A}_1) = \exp[i\pi \nu_{\mathrm{pl}}(\gamma)] \qquad (S16)$$

and equivalently,

$$\nu_{\mathrm{pl}}(\gamma) = I_{\mathrm{ph}}(\gamma_1) \bmod 2 \qquad (S17)$$

This result reveals that: (1) in the trivial twist phase of the major axis with $\nu_{\mathrm{pl}}(\gamma) = 0$, the number of C lines on one side of Π enclosed by the semi-loop γ is even; (2) in the Möbius twist phase of the major axis with $\nu_{\mathrm{pl}}(\gamma) = 1$, the number of C lines on one side of Π enclosed by γ is odd. This relation is illustrated in Fig. S3(B, C) for nontrivial and trivial semi-loops, respectively.

We also note that the arc connecting the two terminal points can be arbitrarily selected. For two selections s_1 and s_2 (see Fig. S3A), the corresponding closed loops are $\gamma_1 = \gamma \circ s_1$ and $\gamma_2 = \gamma \circ s_2$. Their phase indices can be different, providing that the closed path $s_2^{-1} \circ s_1$ encircles some V points in the plane Π:

$$I_{\mathrm{ph}}(\gamma_1) = I_{\mathrm{ph}}(\gamma_2) + I_{\mathrm{ph}}(s_2^{-1} \circ s_1) \qquad (S18)$$

However, since $s_2^{-1} \circ s_1 \subset \Pi$ must carry an even phase index, the parity of the phase index along $\gamma \circ s$ for arbitrary s are always invariant

$$I_{\mathrm{ph}}(\gamma_1) = I_{\mathrm{ph}}(\gamma_2) \bmod 2 \tag{S19}$$

which is consistent with Eq. (S17) and confirms that the topological classification along the semi-loops is well-defined.

Topological indices of the central C lines protected by generalized rotational symmetry

In this section, we examine the minimal stable charges of the C lines along y axis protected by different discrete rotational symmetries $\bar{C}_n$:

$$R\left(\frac{2\pi}{n}\right)\mathbf{H}\left(R\left(-\frac{2\pi}{n}\right)\mathbf{r}\right) = e^{-\frac{\mathrm{i}2\pi}{n}}\mathbf{H}(\mathbf{r}) \tag{S20}$$

Consider a circle, c, which is centered on the y axis and has the positive normal direction along $+\hat{\mathbf{y}}$. In terms of the n-fold generalized rotational symmetry $\bar{C}_n$, the scalar field $\Psi = \mathbf{H}\cdot\mathbf{H}$ satisfies $\Psi\left(R\left(\frac{2\pi}{n}\right)\mathbf{r}\right) = e^{\frac{\mathrm{i}4\pi}{n}}\Psi(\mathbf{r})$. Using this relation, we obtain the $n\mathbb{Z}$-quantized phase index along the circle c:

$$\begin{aligned} I_{\mathrm{ph}}(c) &= \frac{1}{2\pi}\oint d\mathrm{Arg}\Psi(R(\phi)\mathbf{r}_0) = \frac{n}{2\pi}\int_0^{\frac{2\pi}{n}} d\,\phi\,\partial_\phi \mathrm{Arg}\Psi(R(\phi)\mathbf{r}_0) \\ &= \frac{n}{2\pi}\left[\mathrm{Arg}\Psi\left(R\left(\frac{2\pi}{n}\right)\mathbf{r}_0\right) - \mathrm{Arg}\Psi(\mathbf{r}_0)\right] = \frac{n}{2\pi}\left[\frac{4\pi}{n} + 2m\pi\right] \\ &= 2 + nm \in \{\cdots, 2-2n, 2-n, 2, 2+n, 2+2n, \cdots\} \end{aligned} \tag{S21}$$

where $\mathbf{r}_0$ is an arbitrary point on the circle. As we have analyzed in the main text, the minimal charge of $I_{\mathrm{ph}}(c)$ determines the polarization and phase indices of the C line along the y axis $I_{\mathrm{pl}} = +I_{\mathrm{ph}}^{\min}/2$. Therefore, for $n = 1,2$, $I_{\mathrm{pl}} = 0$, indicating the y axis is not a topologically stable C line; for $n = 3$, $I_{\mathrm{pl}} = -1$; for $n = 4$, $I_{\mathrm{pl}} = \pm 1$, indicating the central C line is second order with either positive or negative charges; for $n \geq 5$, the central C lines is always of positive second order with $I_{\mathrm{pl}} = +1$.

In what follows, we will demonstrate this general relation between the order of the central C line and the order of the discrete generalized rotational symmetry via the perturbation analysis near the y axis. For convenience, we adopt the circular basis of transverse coordinates $r_\pm = z \pm \mathrm{i}x = re^{\pm\mathrm{i}\vartheta}$ which satisfies $R(\phi)r_\pm = e^{\pm\mathrm{i}\phi}r_\pm$, and expand the field in the transverse plane (i.e., *xoz*-plane) of a fixed point $\mathbf{r}_0 = y_0\hat{\mathbf{y}}$ on the y axis***:***

$$\begin{aligned} \mathbf{H}(\mathbf{r}) &= \mathbf{H}(r_+, r_-, y_0) \\ &= \mathbf{h}^{(0)} + (\mathbf{h}_+^{(1)}r_+ + \mathbf{h}_-^{(1)}r_-) + (\mathbf{h}_{++}^{(2)}r_+^2 + \mathbf{h}_{--}^{(2)}r_-^2 + \mathbf{h}_\pm^{(2)}r_+r_-) + \mathcal{O}(r^3) \end{aligned} \tag{S22}$$

where $\mathbf{h}^{(0)} = \mathbf{H}(\mathbf{r}_0) = \mathbf{R}$. According to Eq. (S20), the first order terms satisfy

$$e^{\mp\frac{\mathrm{i}2\pi}{n}}R\left(\frac{2\pi}{n}\right)\mathbf{h}_\pm^{(1)} = e^{-\frac{\mathrm{i}2\pi}{n}}\mathbf{h}_\pm^{(1)}$$

$$\Rightarrow \begin{cases} R\left(\frac{2\pi}{n}\right)\mathbf{h}_+^{(1)} = \mathbf{h}_+^{(1)} & \Rightarrow \quad \mathbf{h}_+^{(1)} = a_1\hat{\mathbf{y}} \\ R\left(\frac{2\pi}{n}\right)\mathbf{h}_-^{(1)} = e^{-\frac{\mathrm{i}4\pi}{n}}\mathbf{h}_-^{(1)} & \Rightarrow \quad \mathbf{h}_-^{(1)} = a_2\mathbf{L}\ (n=3)\ \text{ or }\ \mathbf{0}\ (n \geq 4) \end{cases} \tag{S23}$$

The second order terms satisfy

$$e^{-\frac{\mathrm{i}4\pi}{n}}R\left(\frac{2\pi}{n}\right)\mathbf{h}_{++}^{(2)} = e^{-\frac{\mathrm{i}2\pi}{n}}\mathbf{h}_{++}^{(2)} \quad \Rightarrow \quad R\left(\frac{2\pi}{n}\right)\mathbf{h}_{++}^{(2)} = e^{\frac{\mathrm{i}2\pi}{n}}\mathbf{h}_{++}^{(2)} \quad \Rightarrow \quad \mathbf{h}_{++}^{(2)} = a_3\mathbf{L} \tag{S24}$$

$$e^{\frac{i4\pi}{n}}R\left(\frac{2\pi}{n}\right)\mathbf{h}_{--}^{(2)}=e^{-\frac{i2\pi}{n}}\mathbf{h}_{--}^{(2)}\quad\Rightarrow\quad R\left(\frac{2\pi}{n}\right)\mathbf{h}_{--}^{(2)}=e^{-\frac{i6\pi}{n}}\mathbf{h}_{--}^{(2)}$$

$$\Rightarrow\quad \mathbf{h}_{--}^{(2)}=a_4\hat{\mathbf{y}}\ (n=3)\ \text{ or } a_4\mathbf{L}\ (n=4)\ \text{ or }\ \mathbf{0}\ (n\geq 5)\tag{S25}$$

$$R\left(\frac{2\pi}{n}\right)\mathbf{h}_{\pm}^{(2)}=e^{-\frac{i2\pi}{n}}\mathbf{h}_{\pm}^{(2)}\quad\Rightarrow\quad\mathbf{h}_{\pm}^{(2)}=a_5\mathbf{R}\tag{S26}$$

Therefore, we obtain the perturbative magnetic field near the y axis under the constrains of different $\bar{C}_n$ symmetry.

$\bar{C}_3$ symmetry case:

$$\begin{aligned}\mathbf{H}(r_+,r_-,y_0)&=\mathbf{R}+(a_1\hat{\mathbf{y}}r_++a_2\mathbf{L}r_-)+(a_3\mathbf{L}r_+^2+a_4\hat{\mathbf{y}}r_-^2+a_5\mathbf{R}r_+r_-)+\mathcal{O}(r^3)\\&=\mathbf{R}+r\left(a_1\hat{\mathbf{y}}e^{i\vartheta}+a_2\mathbf{L}e^{-i\vartheta}\right)+r^2\left(a_3\mathbf{L}e^{i2\vartheta}+a_4\hat{\mathbf{z}}e^{-i2\vartheta}+a_5\mathbf{R}\right)+\mathcal{O}(r^3)\end{aligned}\tag{S27}$$

$$\Rightarrow\qquad\Psi(r,\vartheta)=\mathbf{H}(r,\vartheta)\cdot\mathbf{H}(r,\vartheta)=2a_2re^{-i\vartheta}+\mathcal{O}(r^2)\tag{S28}$$

Therefore, by $\bar{C}_3$ symmetry, the C-line along the y axis takes the stable charge $I_{\text{ph}}=-1$.

$\bar{C}_4$ symmetry case:

$$\begin{aligned}\mathbf{H}(r_+,r_-,y_0)&=\mathbf{R}+a_1\hat{\mathbf{y}}r_++(a_3\mathbf{L}r_+^2+a_4\mathbf{L}r_-^2+a_5\mathbf{R}r_+r_-)+\mathcal{O}(r^3)\\&=\mathbf{R}+ra_1\hat{\mathbf{y}}e^{i\vartheta}+r^2\left(a_3\mathbf{L}e^{i2\vartheta}+a_4\mathbf{L}e^{-i2\vartheta}+a_5\mathbf{R}\right)+\mathcal{O}(r^3)\end{aligned}\tag{S29}$$

$$\Rightarrow\quad\Psi(r,\vartheta)=\mathbf{H}(r,\vartheta)\cdot\mathbf{H}(r,\vartheta)=r^2\left[(a_1^2+a_3)e^{2i\vartheta}+a_4e^{-2i\vartheta}\right]+\mathcal{O}(r^3)\tag{S30}$$

Therefore, we conform that $\bar{C}_4$ symmetry requires the charge of the central C line takes either $I_{\text{ph}}=2$ (if $|a_1^2+a_3|>|a_4|$) or $I_{\text{ph}}=-2$ (if $|a_1^2+a_3|<|a_4|$). When $|a_1^2+a_3|=|a_4|$, we have $\Psi\left(r,\frac{1}{4}(\pi+\text{Arg}(\frac{a_1^2+a_3}{a_4}))+\frac{n\pi}{2}\right)=0$ which indicates four first-order C lines grow out from the central C-line at the angles $\phi_n=\frac{1}{4}(\pi+\text{Arg}(\frac{a_1^2+a_3}{a_4}))+\frac{n\pi}{2}$, $(n=0,1,2,3)$.

$\bar{C}_n$ $(n\geq 5)$ symmetry case:

$$\begin{aligned}\mathbf{H}(r_+,r_-,y_0)&=\mathbf{R}+a_1\hat{\mathbf{y}}r_++(a_3\mathbf{L}r_+^2+a_5\mathbf{R}r_+r_-)+\mathcal{O}(r^3)\\&=\mathbf{R}+ra_1\hat{\mathbf{y}}e^{i\vartheta}+r^2\left(a_3\mathbf{L}e^{i2\vartheta}+a_5\mathbf{R}\right)+\mathcal{O}(r^3)\end{aligned}\tag{S31}$$

$$\Rightarrow\quad\Psi(r,\vartheta)=\mathbf{H}(r,\vartheta)\cdot\mathbf{H}(r,\vartheta)=r^2(a_1^2+a_3)e^{2i\vartheta}+\mathcal{O}(r^3)\tag{S32}$$

Therefore, as long as the degree of the $\bar{C}_n$ symmetry $n\geq 5$, the central C line always takes the charge $I_{\text{ph}}=+2$.

The minimal charge of the central C line protected by $\bar{C}_n$ symmetry also determines the stable polarization patterns in the vicinity of the central axis. In Fig. S4, using Eqs. (S27), (S29) and (S31), we depict four representative structures of the major axis streamlines in the transverse xoz-plane near the central C line in the presence of different $\bar{C}_n$ symmetries. It can be seen that $\bar{C}_n$ symmetry requires the patterns of the major axis are C_n symmetric. For example, the three-pointed star pattern with $I_{\text{pl}}=-1/2$ is the only possible C_3 symmetric configuration with $|I_{\text{pl}}|=1/2$, and all other C_3 symmetric singular patterns have higher $|I_{\text{pl}}|$ and cannot stably exit without more strict symmetry constraints. Similarly, all patterns of $|I_{\text{pl}}|=1/2$ are incompatible with any $\bar{C}_n$ $(n\geq 4)$ symmetry. The spiral vortex ($I_{\text{pl}}=1$) and the saddle ($I_{\text{pl}}=-1$) are the only two patterns with $|I_{\text{pl}}|=1$ admitted by $\bar{C}_4$ symmetry. And for $\bar{C}_{n\geq 5}$ (including the cylindrical case), the spiral vortex is the pattern with minimal polarization index and compatible with the rotational symmetry. When the chirality of incident wave is changed to be left-handed, the generalized rotational symmetries change to $R(\frac{2\pi}{n})\mathbf{H}(R(-\frac{2\pi}{n})\mathbf{r})=e^{+\frac{i2\pi}{n}}\mathbf{H}(\mathbf{r})$, which also requires the patterns of major axis near the central axis are C_n symmetric. Consequently, the minimal polarization index of the

central C line is irrelevant to the chirality of the circularly polarized incident field but only depends on the rotation symmetry of the scatters.

Polarization singularities in metal structures excited by vector Bessel beams

The birth of PSs on the metal structures is protected by the Poincaré-Hopf theorem which has nothing to do with the type of the incident wave, as long as the fields concerned are tangent. Other exciting sources can only change the local distributions of those PSs, but their global properties are fully source-independent: the index sum of the PSs must be equal to the Euler characteristic of the structures. To further confirm this independence, here we study the PSs emerging in the interactions between metal structures and structured vector light beams. Specifically, we consider a circularly-polarized vector Bessel beam impinging upon a metal structure with cylindrical symmetry (e.g., a sphere or a torus) and the structure's axis is coaxial with the incident beam. The electric field of a right-handed circularly polarized n-th order Bessel beam propagating along the z axis is (59):

$$\mathbf{E}_{\rm in}(\mathbf{r}) = \omega\mu_0 k_z\left[\mathrm{i}(\hat{\mathbf{x}}+\mathrm{i}\hat{\mathbf{y}})\,J_n(k_t r)e^{in\vartheta} - \hat{\mathbf{z}}\,J_{n+1}(k_t r)e^{\mathrm{i}(n+1)\vartheta}\right]e^{-\mathrm{i}k_z z} \quad \text{(S33)}$$

where (r,ϑ,z) defines the cylindrical coordinates; $\omega\,,k_t\,,k_z$ denote the angular frequency, transverse and longitudinal wavenumbers, respectively; and $J_n(k_t r)$ is the n-th order Bessel function of the first kind. Apparently, the incident field observes the generalized cylindrical symmetry $\bar{C}_\infty(n)$: $R(\phi)\mathbf{E}_{\rm in}(R(-\phi)\mathbf{r}) = e^{-\mathrm{i}(n+1)\phi}\mathbf{E}_{\rm in}(\mathbf{r})$. That is, an arbitrary rotation $R(\phi)$ along the z-axis would induce an additional phase $-(n+1)\phi$ for the field, which is exactly proportional to the total angular momentum quantum number $(n+1)$. Here $-n\phi$ and $-\phi$ are induced by the orbital and spin components, respectively. For $n=0$, the symmetry is identical to the case of a circularly polarized incident plane wave, which has already been discussed in the main text. Since the magnetic field of the Bessel beam satisfies $\mathbf{H}_{\rm in} = \frac{1}{\mathrm{i}\omega\mu_0}\nabla\times\mathbf{E}_{\rm in}$, it obeys the same symmetry as the electric field: $R(\phi)\mathbf{H}_{\rm in}(R(-\phi)\mathbf{r}) = \frac{1}{\mathrm{i}\omega\mu_0}\nabla\times[R(\phi)\mathbf{E}_{\rm in}(R(-\phi)\mathbf{r})] = e^{-\mathrm{i}(n+1)\phi}\mathbf{H}_{\rm in}(\mathbf{r})$. The symmetry of the metal structure and the incident beam guarantees that the distribution of the total magnetic field also observes $\bar{C}_\infty(n)$-symmetry:

$$R(\phi)\mathbf{H}(R(-\phi)\mathbf{r}) = e^{-\mathrm{i}(n+1)\phi}\mathbf{H}(\mathbf{r}) \quad \text{(S34)}$$

On the z axis, $R(\phi)\mathbf{H}(z\hat{\mathbf{z}}) = e^{-\mathrm{i}(n+1)\phi}\mathbf{H}(z\hat{\mathbf{z}})$ indicates that $\mathbf{H}(z\hat{\mathbf{z}})$ must be either null or a nontrivial eigenvector of $R(\phi)$ with eigenvalue $e^{-\mathrm{i}(n+1)\phi}$. However, since a general rotation matrix $R(\phi)$ only has three eigenvalues $e^{\pm\mathrm{i}\phi}$ and 1, $e^{-\mathrm{i}(n+1)\phi}$ cannot be an eigenvalue of $R(\phi)$ unless $n=0$, -1 or -2, which leads to the conclusions: (1) for the 0-order and -2-order Bessel beams with total angular momentum quantum number $(n+1)=\pm1$, the total magnetic fields along the z-axis form a C line of right- and left-handedness $\mathbf{H}(z\hat{\mathbf{z}}) \propto \frac{1}{\sqrt{2}}(\hat{\mathbf{x}}\pm\mathrm{i}\hat{\mathbf{y}})$, respectively; (2) for the -1-order Bessel beam with total angular momentum quantum number $n+1=0$, the total magnetic fields along the z-axis form a L line of longitudinal linear polarization and vanishing transverse fields; (3) for Bessel beams of other orders, the central z-axis must be a V line with vanishing total magnetic field. Similar conclusions can also be drawn for incident Bessel beams of left-handed circular polarization, as parity symmetry secures the formations of C line and L line along the z-axis for $n=0,+2$ and $n=+1$, respectively.

As argued above, for a spherical metal structure, the total magnetic fields on the north and south poles ($x=y=0$) of the sphere must either form two C points ($n=0$ or -2) or form two V points ($n\neq0,-2$) under the excitation of the right-handed circularly-polarized Bessel beams. And the

$\bar{C}_\infty(n)$ symmetry can further determine the winding of the polarization major axis and the phase of the magnetic field $\mathbf{H}(\mathbf{r}) = (\mathbf{A}(\mathbf{r}) + \mathrm{i}\mathbf{B}(\mathbf{r}))e^{\mathrm{i}\theta(\mathbf{r})}$around the poles:

$$R(\phi)\mathbf{H}(R(-\phi)\mathbf{r}) = R(\phi)\big(\mathbf{A}(R(-\phi)\mathbf{r}) + \mathrm{i}\mathbf{B}(R(-\phi)\mathbf{r})\big)e^{\mathrm{i}\theta(R(-\phi)\mathbf{r})}$$
$$= e^{-\mathrm{i}(n+1)\phi}\mathbf{H}(\mathbf{r}) = (\mathbf{A}(\mathbf{r}) + \mathrm{i}\mathbf{B}(\mathbf{r}))e^{\mathrm{i}\theta(\mathbf{r})-\mathrm{i}(n+1)\phi}$$
$$\Rightarrow \begin{cases} R(\phi)\mathbf{A}(R(-\phi)\mathbf{r}) = \mathbf{A}(\mathbf{r}) \\ \theta(R(-\phi)\mathbf{r}) = \theta(\mathbf{r}) - (n+1)\phi \end{cases} \qquad (S35)$$

The first relation about $\mathbf{A}(\mathbf{r})$ shows that the major axes always distribute in an azimuthally symmetric pattern around the poles, irrespective of the order of the Bessel beams. Therefore, two polarization singularities locate at the poles with fixed polarization indices $I_{\mathrm{pl}} = +1$, being consistent with the Poincaré-Hopf theorem: $\sum I_{\mathrm{pl}} = 1 + 1 = +2 = \chi$ for all orders of incident Bessel beams. The second relation of Eq. (S35) indicates that the phase indices of these two poles are decided by the total angular momentum quantum number of the incident Bessel beam:

$$I_{\mathrm{ph}} = \frac{1}{2\pi}\oint d\phi\, \partial_\phi \mathrm{Arg}[\Psi(R(\phi)\mathbf{r})] = \frac{1}{2\pi}\oint d\phi\, \partial_\phi \mathrm{Arg}[2\theta(R(\phi)\mathbf{r})]$$
$$= \frac{1}{\pi}\oint d\phi\, \partial_\phi \mathrm{Arg}[\theta(\mathbf{r}_0) + (n+1)\phi] = 2(n+1) \qquad (S36)$$

When $n = 0$ or -2, the polarization and phase indices of the two C points satisfy the aforementioned relation (S8): $2I_{\mathrm{pl}} = \mathrm{sign}(\hat{\mathbf{z}} \cdot \mathbf{S})I_{\mathrm{ph}}$. However, for the cases of $n \neq 0, -2$, the relation between the two indices of the two V points has to be modified as $2I_{\mathrm{pl}} = I_{\mathrm{ph}} \bmod 2$. This is natural, since introducing a vortex phase term $e^{in\vartheta}$ around a V point would change I_{ph} to $I_{\mathrm{ph}} + 2n$, with I_{pl} being invariant (the polarization ellipses have nothing to do with overall phase of the field). In contrast, it is not possible to introduce such a phase term around a C point, as it would break the continuity of the field at the C point.

To verify our theory, we numerically simulated the PSLs emerge on a sphere and a torus excited by right-handed circularly polarized Bessel beams of the orders $n = -2, -1, 0, +1, +2$, respectively. The results are summarized in Fig. S5 and Fig. S6. For the sphere case in Fig. S5, we notice that two V lines grow from the north and south poles of the spherical surface when $n = +1, +2$; two C lines grow from the poles of the surface when $n = -2, 0$; two L lines with magnetic field polarized along *z* direction grow from the surface when $n = -1$ (in this case, the two ends of the L lines on the sphere are still two V points since the *z*-component of the magnetic field must vanish on the surface). Importantly, since in each case only the two poles on the sphere are PSs, and they have the same indices $I_{\mathrm{pl}} = +1$, thus the index sum always satisfies the Poincaré-Hopf theorem for sphere $\sum I_{\mathrm{pl}} = \chi = 2$. Meanwhile, the simulated phase vortices on the sphere in Fig. S5 also corroborate the relation between the phase index of the poles and the order of the Bessel beams: $I_{\mathrm{ph}} = 2(n+1)$. For the torus case in Fig. S6, the results are similar, and the only difference is that the PSLs thread through the hole of the torus. The absence of PSs on the torus in all cases also confirms the Poincaré-Hopf theorem for torus $\sum I_{\mathrm{pl}} = \chi = 0$.

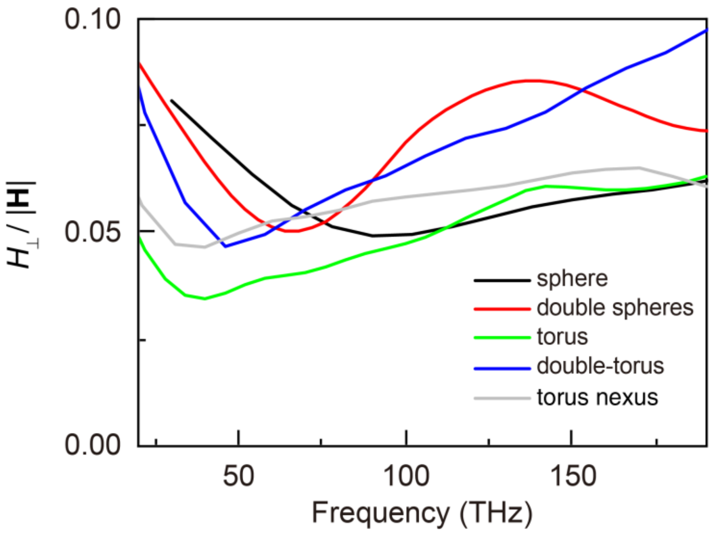

Fig. S1. Normal component of the total magnetic field near the surface of metal structures with different genus. The normal component is normalized by the amplitude of the magnetic field and is averaged over the structural surfaces.

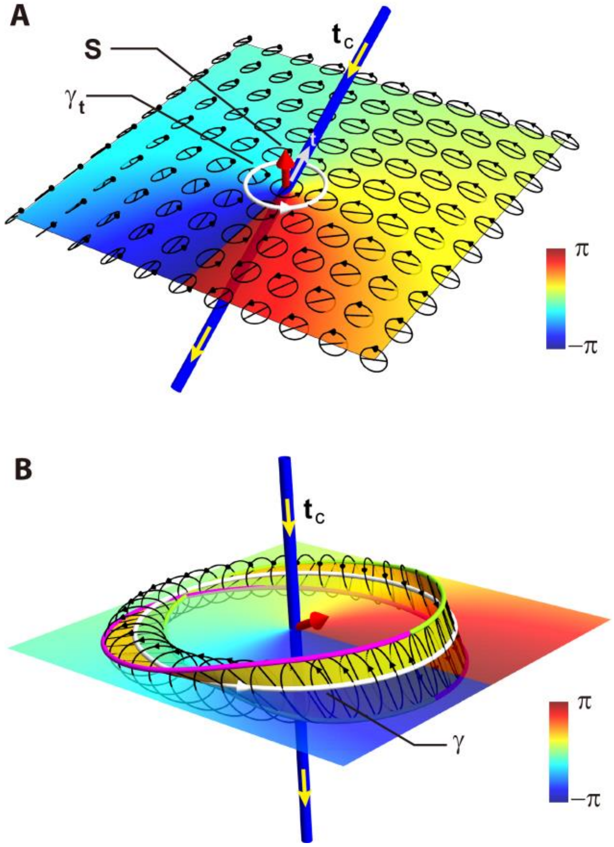

Fig. S2. Schematic of the relations between local polarization index, global polarization index, and phase index around a C line. (**A**) Phase vortex and polarization ellipses lying on the plane perpendicular to the magnetic spin **S** (red arrow) of the central C point on a C line. (**B**) The Möbius strip of polarization ellipses along a finite loop $\boldsymbol{\gamma}$ (white circle) around a C line.

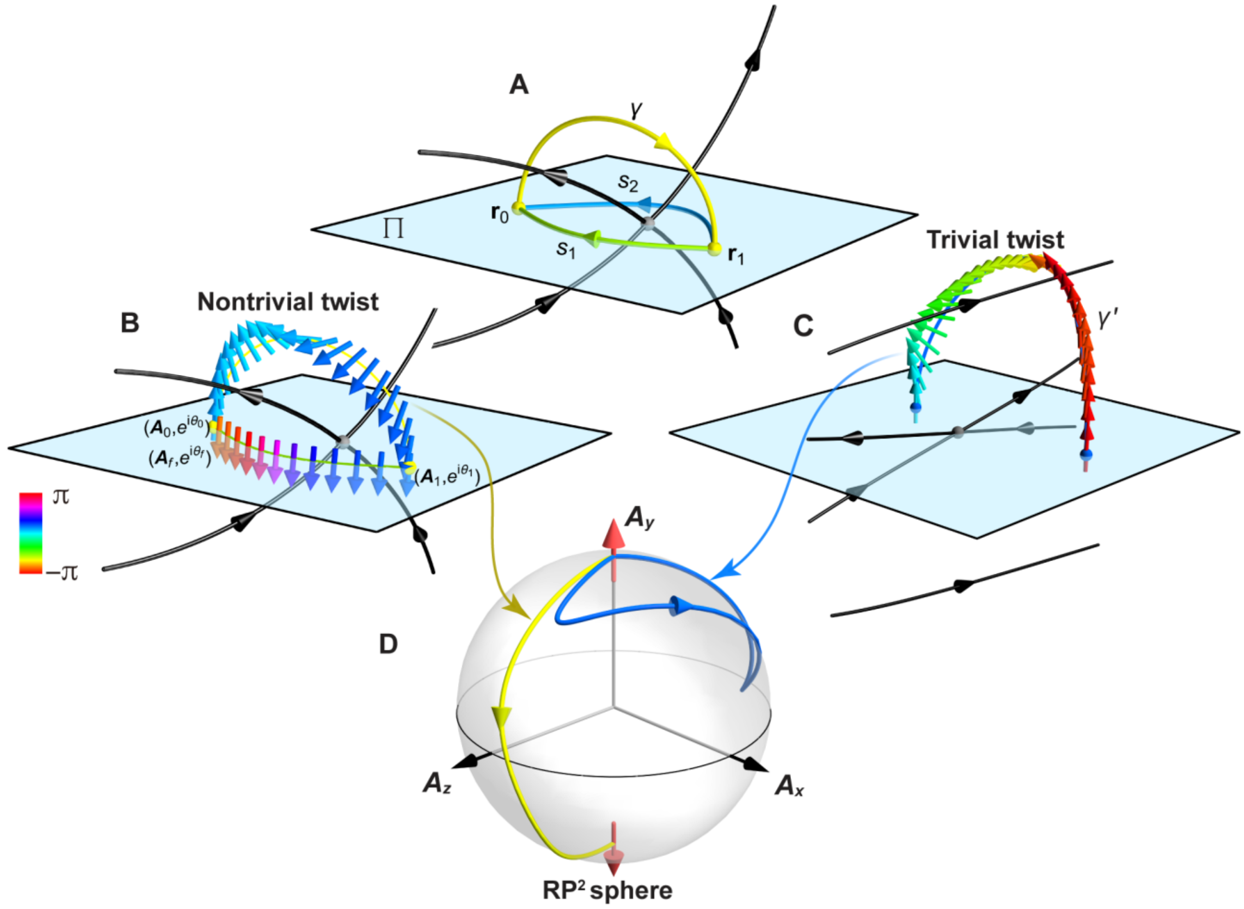

Fig. S3. $\mathbb{Z}_2$ **topology on a semi-loop** $\boldsymbol{\gamma}$ **terminated on the mirror plane Π**. The back lines in (**A**-**C**) denote mirror-partner C lines and their crossings are V points in Π, where the arrows on the C lines shown their positive directions. The colored arrows in (**B**, **C**) denote the polarization major axes along the loop $\gamma_1 = \gamma \circ s_1$ and γ', and the color of the arrows displays the phase angle θ. (**D**) Nontrivial and trivial trajectories on the $\mathbb{R}P^2$ sphere corresponding to the Mobius and trivial twists of the major axis along the semi-loops γ and γ' in (**B**) and (**C**), respectively.

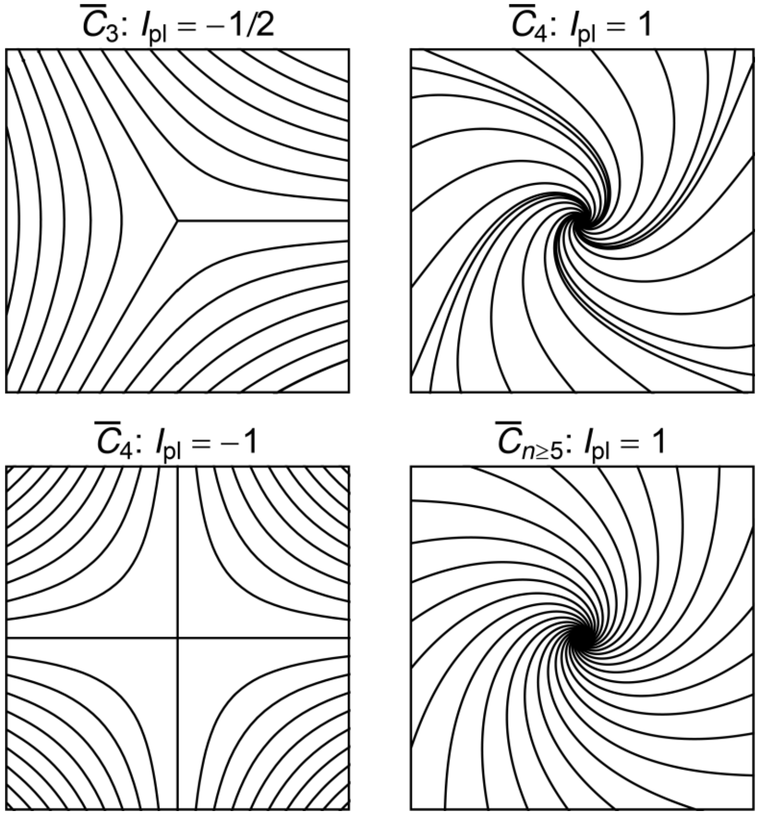

Fig. S4. **Streamlines of major axis in the transverse plane near the stable central C points protected by $\overline{C}_n$ symmetries.** The corresponding symmetry and polarization index are labeled above each pattern of streamlines.

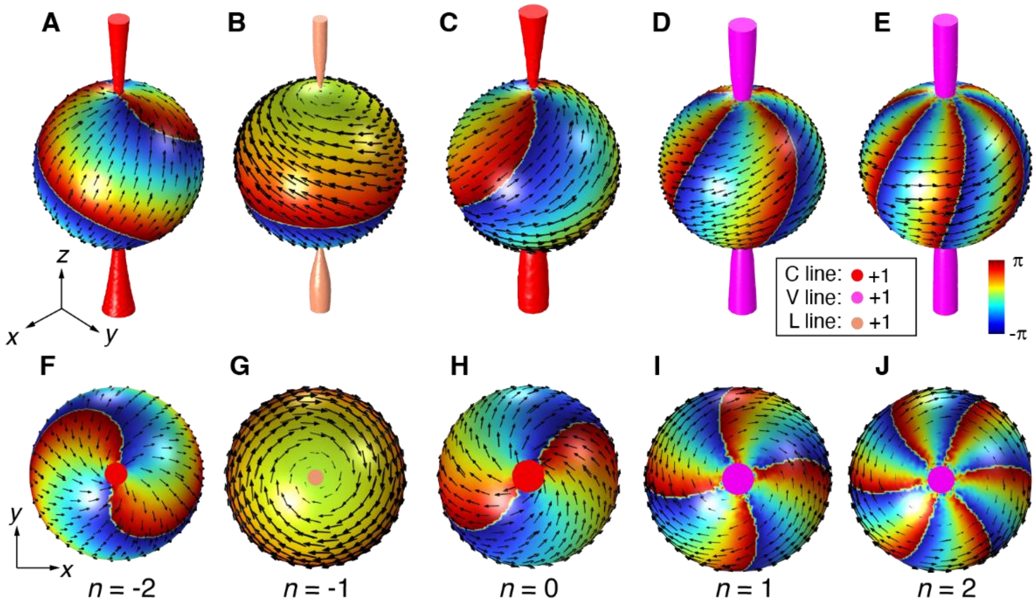

Fig. S5. **Polarization singularity lines for total magnetic field of a sphere excited by circularly polarized Bessel beams**. The color denotes Arg($\mathbf{H} \cdot \mathbf{H}$) and the arrows denote the polarization major axis vector $\mathbf{A}$. The red/magenta/orange lines denote different sorts of PSLs. The excitation frequency is 105 THz.

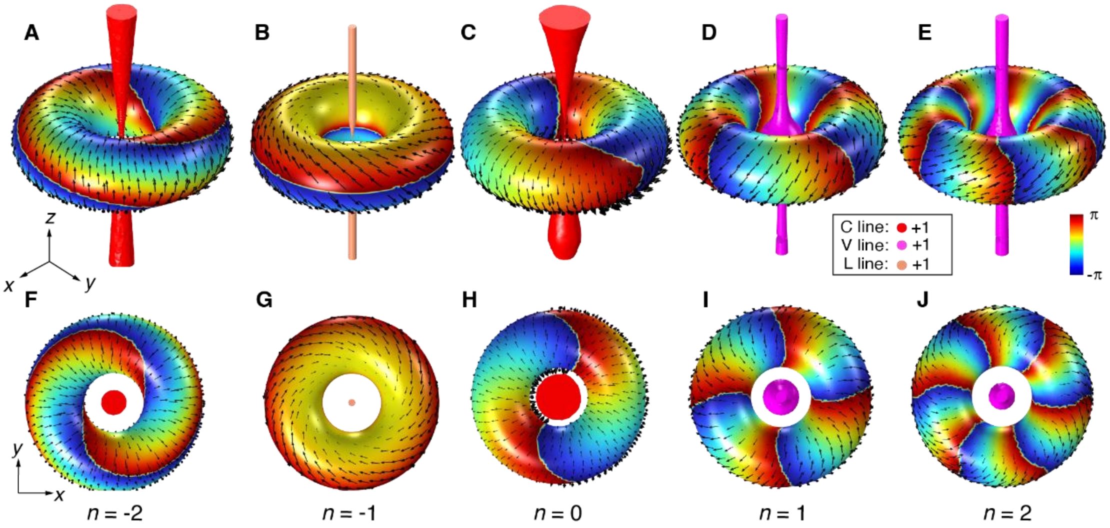

Fig. S6. Polarization singularity lines for total magnetic fields of a torus excited by circularly polarized Bessel beams. The color denotes $\mathrm{Arg}(\mathbf{H}\cdot\mathbf{H})$ and the arrows denote the polarization major axis vector **A**. The red/magenta/orange lines denote different sorts of PSLs. The excitation frequency is 105 THz.

REFERENCES AND NOTES

1. T. Ozawa, H. M. Price, A. Amo, N. Goldman, M. Hafezi, L. Lu, M. C. Rechtsman, D. Schuster, J. Simon, O. Zilberberg, I. Carusotto, Topological photonics. *Rev. Mod. Phys.* **91**, 015006 (2019).

2. M. V. Berry, M. R. Dennis, Knotted and linked phase singularities in monochromatic waves. *Proc. R. Soc. Lond. A* **457**, 2251–2263 (2001).

3. J. Leach, M. R. Dennis, J. Courtial, M. J. Padgett, Knotted threads of darkness. *Nature* **432**, 165 (2004).

4. M. R. Dennis, R. P. King, B. Jack, K. O'Holleran, M. J. Padgett, Isolated optical vortex knots. *Nat. Phys.* **6**, 118–121 (2010).

5. H. Kedia, I. Bialynicki-Birula, D. Peralta-Salas, W. T. M. Irvine, Tying knots in light fields. *Phys. Rev. Lett.* **111**, 150404 (2013).

6. T. Bauer, P. Banzer, E. Karimi, S. Orlov, A. Rubano, L. Marrucci, E. Santamato, R. W. Boyd, G. Leuchs, Observation of optical polarization Möbius strips. *Science* **347**, 964–966 (2015).

7. H. Larocque, D. Sugic, D. Mortimer, A. J. Taylor, R. Fickler, R. W. Boyd, M. R. Dennis, E. Karimi, Reconstructing the topology of optical polarization knots. *Nat. Phys.* **14**, 1079–1082 (2018).

8. E. Pisanty, G. J. Machado, V. Vicuña-Hernández, A. Picón, A. Celi, J. P. Torres, M. Lewenstein, Knotting fractional-order knots with the polarization state of light. *Nat. Photon.* **13**, 569–574 (2019).

9. D. Sugic, R. Droop, E. Otte, D. Ehrmanntraut, F. Nori, J. Ruostekoski, C. Denz, M. R. Dennis, Particle-like topologies in light. *Nat. Commun.* **12**, 6785 (2021).

10. Y. Shen, Y. Hou, N. Papasimakis, N. I. Zheludev, Supertoroidal light pulses as electromagnetic skyrmions propagating in free space. *Nat. Commun.* **12**, 5891 (2021).

11. A. Zdagkas, C. McDonnell, J. Deng, Y. Shen, G. Li, T. Ellenbogen, N. Papasimakis, N. I. Zheludev, Observation of toroidal pulses of light. *Nat. Photon.* **16**, 523–528 (2022).

12. C. Wan, Q. Cao, J. Chen, A. Chong, Q. Zhan, Toroidal vortices of light. *Nat. Photon.* **16**, 519–522 (2022).

13. J. F. Nye, J. V. Hajnal, The wave structure of monochromatic electromagnetic radiation. *Proc. R. Soc. Lond. A* **409**, 21–36 (1987).

14. R. W. Schoonover, T. D. Visser, Polarization singularities of focused, radially polarized fields. *Opt. Express* **14**, 5733–5745 (2006).

15. J. Peng, W. Liu, S. Wang, Polarization singularities in light scattering by small particles. *Phys. Rev. A* **103**, 023520 (2021).

16. K. S. Grigoriev, N. Y. Kuznetsov, Y. V. Vladimirova, V. A. Makarov, Fine characteristics of polarization singularities in a three-dimensional electromagnetic field and their properties in the near field of a metallic nanospheroid. *Phys. Rev. A* **98**, 063805 (2018).

17. N. Y. Kuznetsov, K. S. Grigoriev, Y. V. Vladimirova, V. A. Makarov, Three-dimensional structure of polarization singularities of a light field near a dielectric spherical nanoparticle. *Opt. Express* **28**, 27293–27299 (2020).

18. W. Chen, Q. Yang, Y. Chen, W. Liu, Global Mie scattering: Polarization morphologies and the underlying topological invariant. *ACS Omega* **5**, 14157–14163 (2020).

19. S. Jia, J. Peng, Y. Cheng, S. Wang, Chiral discrimination by polarization singularities of a metal sphere. *Phys. Rev. A* **105**, 033513 (2022).

20. F. Flossmann, K. R. O'Holleran, M. R. Dennis, M. J. Padgett, Polarization singularities in 2D and 3D speckle fields. *Phys. Rev. Lett.* **100**, 203902 (2008).

21. Y. Zhang, X. Yang, J. Gao, Generation of polarization singularities with geometric metasurfaces. *Sci. Rep.* **9**, 19656 (2019).

22. A. H. Dorrah, F. Capasso, Tunable structured light with flat optics. *Science* **376**, eabi6860 (2022).

23. Y. Zhang, A. Chen, W. Liu, C. W. Hsu, B. Wang, F. Guan, X. Liu, L. Shi, L. Lu, J. Zi, Observation of polarization vortices in momentum space. *Phys. Rev. Lett.* **120**, 186103 (2018).

24. S. Wang, G. Ma, C. T. Chan, Topological transport of sound mediated by spin-redirection geometric phase. *Sci. Adv.* **4**, eaaq1475 (2018).

25. K. Y. Bliokh, M. A. Alonso, M. R. Dennis, Geometric phases in 2D and 3D polarized fields: Geometrical, dynamical, and topological aspects. *Rep. Prog. Phys.* **82**, 122401 (2019).

26. B. Zhen, C. W. Hsu, L. Lu, A. D. Stone, M. Soljačić, Topological nature of optical bound states in the continuum. *Phys. Rev. Lett.* **113**, 257401 (2014).

27. H. M. Doeleman, F. Monticone, W. den Hollander, A. Alù, A. F. Koenderink, Experimental observation of a polarization vortex at an optical bound state in the continuum. *Nat. Photon.* **12**, 397–401 (2018).

28. W. Liu, B. Wang, Y. Zhang, J. Wang, M. Zhao, F. Guan, X. Liu, L. Shi, J. Zi, Circularly polarized states spawning from bound states in the continuum. *Phys. Rev. Lett.* **123**, 116104 (2019).

29. C. Fang, H. Weng, X. Dai, Z. Fang, Topological nodal line semimetals. *Chin. Phys. B* **25**, 117106 (2016).

30. N. P. Armitage, E. J. Mele, A. Vishwanath, Weyl and Dirac semimetals in three-dimensional solids. *Rev. Mod. Phys.* **90**, 015001 (2018).

31. W. Chen, Q. Yang, Y. Chen, W. Liu, Evolution and global charge conservation for polarization singularities emerging from non-Hermitian degeneracies. *Proc. Natl. Acad. Sci. U.S.A.* **118**, e2019578118 (2021).

32. H. Zhou, C. Peng, Y. Yoon, C. W. Hsu, K. A. Nelson, L. Fu, J. D. Joannopoulos, M. Soljačić, B. Zhen, Observation of bulk Fermi arc and polarization half charge from paired exceptional points. *Science* **359**, 1009–1012 (2018).

33. H. Shen, B. Zhen, L. Fu, Topological band theory for non-Hermitian Hamiltonians. *Phys. Rev. Lett.* **120**, 146402 (2018).

34. Z. Yang, A. P. Schnyder, J. Hu, C.-K. Chiu, Fermion doubling theorems in two-dimensional non-Hermitian systems for Fermi points and exceptional points. *Phys. Rev. Lett.* **126**, 086401 (2021).

35. K. Wang, A. Dutt, C. C. Wojcik, S. Fan, Topological complex-energy braiding of non-Hermitian bands. *Nature* **598**, 59–64 (2021).

36. P. Biagioni, J.-S. Huang, B. Hecht, Nanoantennas for visible and infrared radiation. *Rep. Prog. Phys.* **75**, 024402 (2012).

37. M. Kadic, G. W. Milton, M. van Hecke, M. Wegener, 3D metamaterials. *Nat. Rev. Phys.* **1**, 198–210 (2019).

38. H.-T. Chen, A. J. Taylor, N. Yu, A review of metasurfaces: Physics and applications. *Rep. Prog. Phys.* **79**, 076401 (2016).

39. I. Freund, Optical Möbius strips, twisted ribbons, and the index theorem. *Opt. Lett.* **36**, 4506–4508 (2011).

40. N. Yu. Kuznetsov, K. S. Grigoriev, V. A. Makarov, Topology of polarization-ellipse strips in the light scattered by a dielectric nanosphere. *Phys. Rev. A* **104**, 043505 (2021).

41. M. V. Berry, Index formulae for singular lines of polarization. *J. Opt. A: Pure Appl. Opt.* **6**, 675–678 (2004).

42. T. Needham, *Visual Differential Geometry and Forms: A Mathematical Drama in Five Acts* (Princeton University Press, 2021).

43. C. C. Wojcik, X.-Q. Sun, T. Bzdušek, S. Fan, Homotopy characterization of non-Hermitian Hamiltonians. *Phys. Rev. B* **101**, 205417 (2020).

44. I. Freund, Optical Möbius strips in three-dimensional ellipse fields: I. Lines of circular polarization. *Opt. Commun.* **283**, 1–15 (2010).

45. E. J. Galvez, I. Dutta, K. Beach, J. J. Zeosky, J. A. Jones, B. Khajavi, Multitwist Möbius strips and twisted ribbons in the polarization of paraxial light beams. *Sci. Rep.* **7**, 13653 (2017).

46. M. V. Berry, M. R. Dennis, R. L. Lee, Polarization singularities in the clear sky. *New J. Phys.* **6**, 162–162 (2004).

47. R. P. Feynman, R. B. Leighton, M. Sands, *The Feynman Lectures on Physics, Vol. III: Quantum Mechanics* (Basic Books, 2011).

48. Q. Yang, W. Chen, Y. Chen, W. Liu, Symmetry protected invariant scattering properties for incident plane waves of arbitrary polarizations. *Laser Photonics Rev.* **15**, 2000496 (2021).

49. W. Ye, Y. Gao, J. Liu, Singular points of polarizations in the momentum space of photonic crystal slabs. *Phys. Rev. Lett.* **124**, 153904 (2020).

50. C. W. Hsu, B. Zhen, J. Lee, S.-L. Chua, S. G. Johnson, J. D. Joannopoulos, M. Soljačić, Observation of trapped light within the radiation continuum. *Nature* **499**, 188–191 (2013).

51. T. Fösel, V. Peano, F. Marquardt, L lines, C points and Chern numbers: Understanding band structure topology using polarization fields. *New J. Phys.* **19**, 115013 (2017).

52. K. Y. Bliokh, F. J. Rodríguez-Fortuño, F. Nori, A. V. Zayats, Spin-orbit interactions of light. *Nat. Photon.* **9**, 796–808 (2015).

53. S. Wang, B. Hou, W. Lu, Y. Chen, Z. Q. Zhang, C. T. Chan, Arbitrary order exceptional point induced by photonic spin–Orbit interaction in coupled resonators. *Nat. Commun.* **10**, 832 (2019).

54. N. Rotenberg, L. Kuipers, Mapping nanoscale light fields. *Nat. Photon.* **8**, 919–926 (2014).

55. S. Wang, G. Zhang, X. Wang, Q. Tong, J. Li, G. Ma, Spin-orbit interactions of transverse sound. *Nat. Commun.* **12**, 6125 (2021).

56. K. Y. Bliokh, M. A. Alonso, D. Sugic, M. Perrin, F. Nori, E. Brasselet, Polarization singularities and Möbius strips in sound and water-surface waves. *Phys. Fluids* **33**, 077122 (2021).

57. R. L. Olmon, B. Slovick, T. W. Johnson, D. Shelton, S.-H. Oh, G. D. Boreman, M. B. Raschke, Optical dielectric function of gold. *Phys. Rev. B.* **86**, 235147(2012).

58. A. Hatcher, *Algebraic Topology* (Cambridge University Press, 2001).

59. Y. Wang, W. Dou, H. Meng, Vector analyses of linearly and circularly polarized Bessel beams using Hertz vector potentials, *Opt. Express* **22**, 7821 (2014).
